# New information on the braincase and inner ear of *Euparkeria capensis* Broom: implications for diapsid and archosaur evolution

**DOI:** 10.1098/rsos.160072

**Published:** 2016-07-13

**Authors:** Gabriela Sobral, Roland B. Sookias, Bhart-Anjan S. Bhullar, Roger Smith, Richard J. Butler, Johannes Müller

**Affiliations:** 1 Departamento de Ecologia e Zoologia, Universidade Federal de Santa Catarina, Florianópolis, SC, Brazil; 2 Departamento de Geologia e Paleontologia, Museu Nacional do Rio de Janeiro, Rio de Janeiro, RJ, Brazil; 3 Museum für Naturkunde Berlin, Leibniz-Institut für Evolutions- und Biodiversitätsforschung, Berlin, Germany; 4 School of Geography, Earth and Environmental Sciences, University of Birmingham, Birmingham, UK; 5 GeoBio-Center, Ludwig-Maximilians-Universität München, München, Germany; 6 Department of Geology and Geophysics and Peabody Museum of Natural History, Yale University, New Haven, CT, USA; 7 Evolutionary Studies Institute, University of the Witwatersrand, Johannesburg, South Africa; 8 Iziko South African Museum, Cape Town, South Africa

**Keywords:** *Euparkeria*, diapsid, archosaur, computer tomography scan, inner ear, braincase

## Abstract

Since its discovery, *Euparkeria capensis* has been a key taxon for understanding the early evolution of archosaurs. The braincase of *Euparkeria* was described based on a single specimen, but much uncertainty remained. For the first time, all available braincase material of *Euparkeria* is re-examined using micro-computed tomography scanning. Contrary to previous work, the parabasisphenoid does not form the posterior border of the fenestra ovalis in lateral view, but it does bear a dorsal projection that forms the anteroventral half of the fenestra. No bone pneumatization was found, but the lateral depression of the parabasisphenoid may have been pneumatic. We propose that the lateral depression likely corresponds to the anterior tympanic recess present in crown archosaurs. The presence of a laterosphenoid is confirmed for *Euparkeria*. It largely conforms to the crocodilian condition, but shows some features which make it more similar to the avemetatarsalian laterosphenoid. The cochlea of *Euparkeria* is elongated, forming a deep cochlear recess. In comparison with other basal archosauromorphs, the metotic foramen is much enlarged and regionalized into vagus and recessus scalae tympani areas, indicating an increase in its pressure-relief mechanism. The anterior semicircular canal is extended and corresponds to an enlarged floccular fossa. These aspects of the braincase morphology may be related to the development of a more upright posture and active lifestyle. They also indicate further adaptations of the hearing system of *Euparkeria* to terrestriality.

## 1. Introduction

Archosauria, a crown group of diapsid reptiles represented today by birds and crocodilians and including the extinct dinosaurs, is highly speciose (with over 9000 species of modern birds and crocodilians [1]) and has been so since its origin in the Late Triassic. Archosaurs filled most terrestrial ecological niches for large-bodied vertebrates for over 150 Myr [2–4], from the Late Triassic to the end of the Cretaceous. The rise of the archosaurs to this position of ecological dominance took place following diversity decline among therapsids, which had previously filled most macroscale terrestrial niches (e.g. [2,4–12]). This faunal transition began at the end of the Permian and continued through the Triassic [4,12,13]. The rise of archosaurs is a landmark terrestrial faunal transition and an outstanding example of an ecological radiation over geological timescales [3].

*Euparkeria capensis* is a small (known individuals reaching approx. 1 m in length [14]) stem archosaur represented by the remains of over 10 individuals collected from a single locality in Subzone B of the *Cynognathus* Assemblage Zone [15,16] (the uppermost biozone of the Burgersdorp Formation and the Beaufort Group), close to Aliwal North, Eastern Cape, South Africa [14,17]. Subzone B is probably Anisian (Middle Triassic) in age [16]. Since its discovery, *Euparkeria* has been considered to be an important taxon for our understanding of the rise and early evolution of archosaurs. *Euparkeria* is nearly universally found to be either the sister taxon to, or a very close relative of, Archosauria in phylogenetic analyses [3,18–28]. For this reason *Euparkeria* is often used as an outgroup in phylogenetic and evolutionary analyses of crown taxa (e.g. [29–41]), allowing the sequence and direction of morphological changes during the radiation of Archosauria to be understood.

Given its phylogenetic position and lack of unique autapomorphies, the morphology of *Euparkeria* has been considered to potentially approach that of the ancestor of Archosauria, and thus may shed light on the early evolution of archosaurs [42]. The gracile, cursorial body plan of *Euparkeria* represents a morphological stage intermediate between more ‘sprawling’ non-archosaurian archosauromorph taxa and fully erect, and often bipedal [12,43,44] crown taxa. Beyond this, *Euparkeria* itself represents a part of the radiation of archosauromorphs, within which the crown radiation is nested. Although often used as a phylogenetic outgroup to Archosauria, *Euparkeria* can also be seen as displaying a relatively derived braincase morphology in comparison to many stem taxa (e.g. relatively high, dorsoventrally elongated parabasisphenoid, elongated semicircular canals, discussed below), representing a continuation of morphological developments which begin further down the archosaur stem.

The braincase of *Euparkeria* was originally described by Ewer [14], based on the holotype (SAM-PK-5867), SAM-PK-7696 and UMZC T.692 (‘Watson's specimen A’; formerly R 527), in a monographic treatment of the taxon. Subsequently, an isolated braincase from specimen SAM-PK-7696 was further acid prepared and was described by Cruickshank [45]. Evans [46] figured this same isolated braincase and used it as a comparator in her treatment of the braincase of *Prolacerta broomi*. Welman [47] figured both SAM-PK-7696 and the braincase of the holotype, which had been further mechanically prepared in the interim. Welman [47] compared the morphology of the braincase of *Euparkeria* to that of birds, dinosaurs and crocodilians, and came to the controversial conclusion that *Euparkeria* was more closely related to birds than to dinosaurs or crocodilians, resurrecting the idea that birds and dinosaurs had separate origins among the ‘thecodonts', a paraphyletic assemblage of stem archosaurs and early pseudosuchians [48]. Gower & Weber [42] thoroughly redescribed the braincase of *Euparkeria*, based primarily on UMZC T.692. In addition to providing a comprehensive reference work, these authors presented evidence refuting the presence or importance of most of the anatomical features used by Welman [47] to link *Euparkeria* to birds to the exclusion of other archosaurs.

Here, we provide a thorough redescription of the osteology of the braincase of *Euparkeria*, building on the work of Gower & Weber [42] and bringing new clarification to points of doubt, documenting new information and confirming areas where our understanding is limited by the material. Although the work of Gower & Weber [42] was thorough, given the material and methods available to the authors, recent advances in computed tomography (CT) allow new insights into the braincase and inner ear anatomy. All material pertaining to the braincase of *Euparkeria* was available for us to examine, and we were able to CT scan the specimen available to Gower & Weber [42] (UMZC T.692), the holotype (SAM-PK-5867), specimen SAM-PK-6047A and the isolated braincase SAM-PK-7696.

**Table 1.** Nomenclature.

?	uncertainty regarding identification	ip	interparietal
aa	anterior ampulla	is	interorbital septum
aip	anterior inferior process of prootic	ld	lateral depression
ap	ascending process of parabasisphenoid	lg.cr	lagenar crest
arts	articular surface	lj	lower jaw
asc	anterior semicircular canal	ls	laterosphenoid
bb	bridge of bone	ls.btr	laterosphenoid buttress
bo	basioccipital	lsc	lateral semicircular canal
bp	basipterygoid process	m	maxilla
bt	basal tuber	md.rd	median ridge
cc	common crus	mf	metotic foramen
cl	cochlea	mpr	median pharyngeal recess
CN I	foramen for cranial nerve I	mx	matrix
CN II	foramen for cranial nerve II	oc	occipital condyle
CN III	foramen for cranial nerve III	op	opisthotic
CN IV	foramen for cranial nerve IV	ov.dp	oval depression
CN V	foramen for cranial nerve V	pa	parietal
CN VI	foramen for cranial nerve VI	pbs	parabasisphenoid
CN VII	foramen for cranial nerve VII	pf	perilymphatic foramen
CN VII_hym_	groove for hyomandibular branch of cranial nerve VII	pp	paroccipital process
CN VII_pal_	groove for palatine branch of cranial nerve VII	pr	prootic
CN XII	foramen for cranial nerve XII	psc	posterior semicircular canal
CN XII_a_	foramen for anterior branch of cranial nerve XII	psa	posterior ampulla
CN XII_p_	foramen for posterior branch of cranial nerve XII	pt	pterygoid
cap	capitate process	ptf	posttemporal fenestra
cp	cultriform process	q	quadrate
cr1	crest 1	rd	ridge
cr2	crest 2	s	suture
ds	dorsum sellae	sd	semilunar depression
eo	exoccipital	st.gr	stapedial groove
f	frontal	so	supraoccipital
fc.pa	facet for parietal	sp	slender process
ff	floccular fossa	st	stapes
fm	foramen magnum	su	sulcus
fo	fenestra ovalis	tc	tensor crest
gr.ga	groove for Gasserian ganglion	tu	tuber
gr	groove	ug	unossified gap
gr.ut	groove marking ventral connection between common crus and utriculus	vcd	vena capitis dorsalis channel
gr.ov.dp.VII	groove connecting oval depression with foramen for cranial nerve VII	ve	vestibule
hf	hypophyseal fossa	vr.op	ventral ramus of the opsithotic
ica	path of internal carotid artery	vt	vertebra

CT scanning allows us to provide additional information on sutures and contacts between elements, as well as details of the internal structures of the braincase and the morphology of the inner ear. Furthermore, we provide thorough documentation of the element generally regarded as a laterosphenoid in *Euparkeria*, describing for the first time its morphology in SAM-PK-5867 and conducting an extensive discussion on its morphology and potential homology.

Our work makes the braincase of *Euparkeria* one of the best-documented early archosauriform braincases and provides a reference point for archosauriform morphologists that will contribute to a growing understanding of the rise and evolutionary radiation of the archosaurs.

## 2. Material and methods

SAM-PK-7696 and UMCZ T.692 (electronic supplementary material, figures S1 and S2) were scanned at the Museum für Naturkunde, Berlin, using a Phoenix|x-ray Nanotom (GE Sensing and Inspection Technologies GmbH, Wunstorf, Germany). The scans comprised a total of 1440 slices, using a tungsten target and a Cu filter of 0.1 mm thickness in modus 0 with averaging 3 and skip 2. The scans of SAM-PK-7696 were reconstructed with the software datos|x-reconstruction v. 1.5.0.22, whereas scans of UMCZ T.692 were reconstructed using datos|x 2 reconstruction v. 2.2.1.739 (both from GE Sensing and Inspection Technologies GmbH, Phoenix|x-ray). Scan settings were as follows - SAM-PK-7696: 80 kV, 250 µA, 1000 ms, 16.34 µm voxel size; UMCZ T.692: 120 kV, 250 µA, 1000 ms, 24.49 µm voxel size.

Specimens SAM-PK-5867 (electronic supplementary material, figure S3) and SAM-PK-6047A were scanned at the Evolutionary Studies Institute (formerly Bernard Price Institute for Palaeontological Research), University of the Witwatersrand. Scanning was conducted with an X Tek HMX ST 225 (Nikon Metrology Inc.), comprising 3000 projections, using a tungsten target with gain 4 and binning 0. Files were reconstructed using CT Pro 3D software (Nikon Metrology, Inc.). Scan settings were as follows - SAM-PK-5867: 70 kV, 140 µA, 1000 ms, 57.50 µm voxel size, 1.8 mm Al filter; SAM-PK-6047A: 120 kV, 95 µA, 2000 ms, 60.10 µm voxel size, 1.2 mm Cu filter.

In addition, four braincases of extant species were scanned at the Museum für Naturkunde Berlin for comparative purposes. Machine settings were the same as described earlier, except 1000 slices were made with the function Fast Scan and no filter (except if stated otherwise). Scan setting were as follows - *Meleagris gallopavo* (ZMB 1793 792): 75 kV, 240 µA, 750 ms, 17.05 µm voxel size; *Sphenodon punctatus* (ROM R9298): 75 kV, 280 µA, 750 ms, 19.44 µm voxel size; *Struthio camelus* (ZMB 2000 2769): 90 kV, 400 µA, 750 ms, 30 µm voxel size; *Osteolaemus tetraspis* (ZMB 23467): 90 kV, 350 µA, 1000 ms, 32.37 µm voxel size and Cu filter.

All scans were post-processed and segmented using VG Studio Max 2.1 and 2.2 (Volume Graphics, Heidelberg, Germany).

## 3. Institutional abbreviations

BP: Evolutionary Studies Institute (formerly Bernard Price Institute for Palaeontological Research), University of the Witwatersrand, Johannesburg, South Africa
NM: National Museum, Bloemfontein, South Africa
PIN: Paleontological Institute of the Russian Academy of Sciences, Moscow, Russia
PVSJ: División de Paleontología, Museo de Ciencias Naturales de la Universidad Nacional de San Juan, Argentina
ROM: Royal Ontario Museum, Toronto, Canada
SAM: Iziko South African Museum, Cape Town, South Africa
UCMP: University of California Museum of Paleontology, Berkeley, USA
UMZC: University Museum of Zoology, University of Cambridge, Cambridge, UK
ZMB: Museum für Naturkunde Berlin, Berlin, Germany
ZPAL: Institute of Paleobiology of the Polish Academy of Sciences, Warsaw, Poland

## 4. Description

### 4.1. Basioccipital

**Figure 1.**
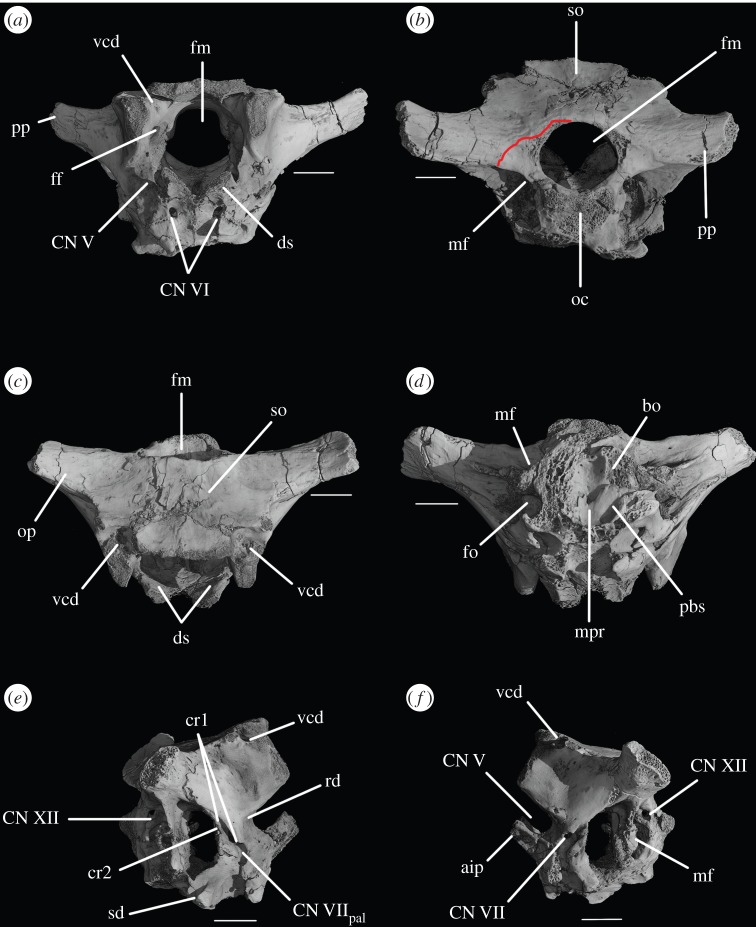
CT reconstruction of the braincase of SAM-PK-7696 in (*a*) anterior, (*b*) posterior, (*c*) dorsal, (*d*) ventral, (*e*) right lateral and (*f*) left lateral views. Red line in (*b*) indicates the suture line between exoccipital and opisthotic/supraoccipital based on CT scans (see figure 6). For abbreviations, see table 1.

**Figure 2.**
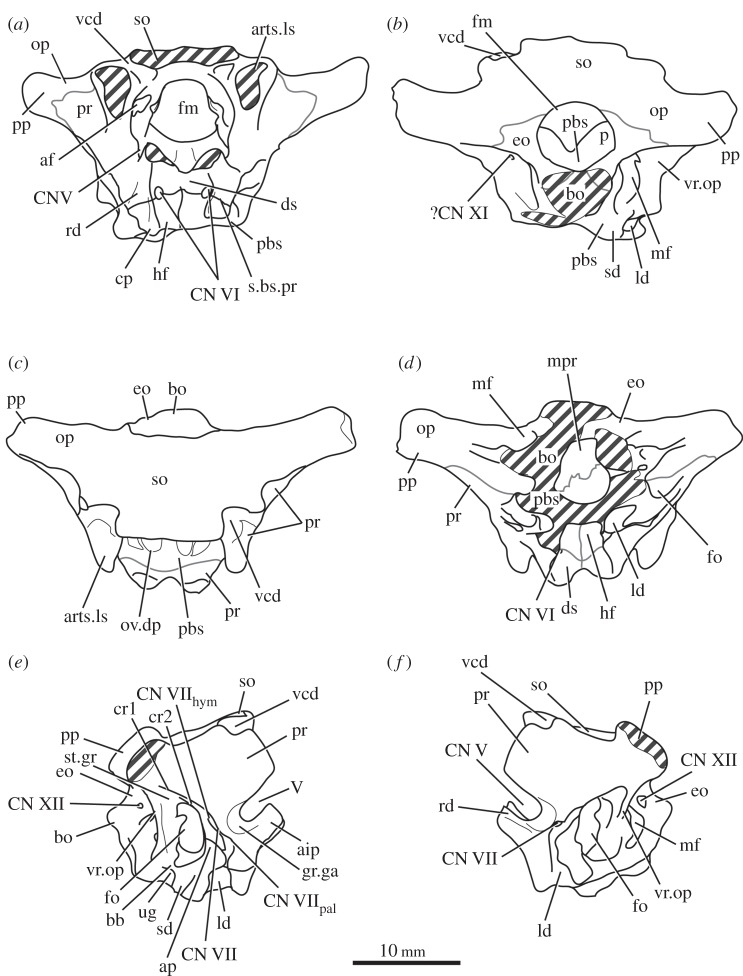
Line drawings of figure 1. Braincase of SAM-PK-7696 in (*a*) anterior, (*b*) posterior, (*c*) dorsal, (*d*) ventral, (*e*) right lateral, and (*f*) left lateral views. For abbreviations, see table 1.

The basioccipital forms the majority of the occipital condyle, with only the dorsolateral corners of the condyle formed by the exoccipitals. The entire occipital condyle (including the exoccipital contribution) is hemispherical, with the dorsal margin being very gently concave in posterior view (figures 1*b*, 7*b* and 11*b*). There is no pronounced ridge delimiting the condyle from the condyle neck (figure 11*b*), unlike in *Dorosuchus neoetus* [49], nor is there a notochordal pit like in *Youngina capensis* [50]. The contribution of the basioccipital to the border of the foramen magnum is very limited, not accounting for more than the middle third of the ventral border of the foramen (figures 1*b*, 6*a* and 11*b*). Thus, the interpretation of Cruickshank [45, fig. 2] (also Gower & Weber [42, fig. 1*b*]) to some extent exaggerated the basioccipital contribution to the foramen magnum. The basioccipital articulates with the exoccipital in a dorsomedial–ventrolateral orientated plane, below the foramen for cranial nerve (CN) XII (figure 6*a,c*).

**Figure 3.**
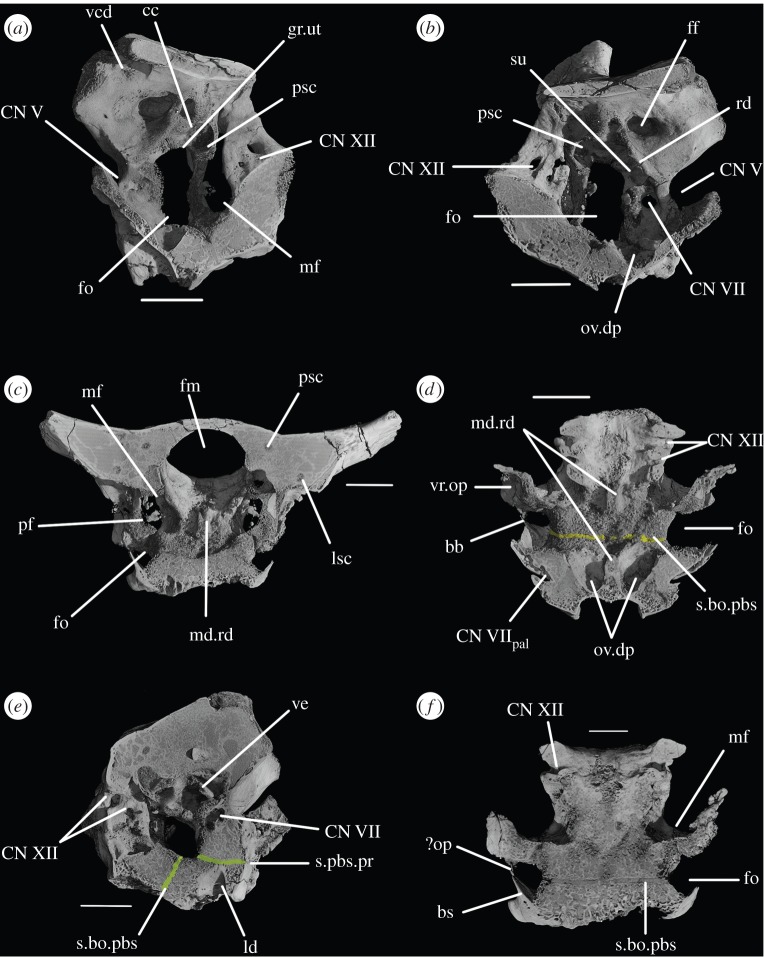
CT reconstructions of the braincase of SAM-PK-7696 (*a*) in right medial view, (*b*) in left medial view, (*c*) in anterior view (only posterior part showing, anterior cut off), (*d*) showing braincase floor in dorsal view, (*e*) in cross section to right of midline through opisthotic, to show basisphenoid contribution to ATR and (*f*) in cross section showing braincase floor in dorsal view, more ventral than (*d*), showing detail of basisphenoid posterior contact with ventral ramus of the opisthotic. For abbreviations, see table 1.

**Figure 4.**
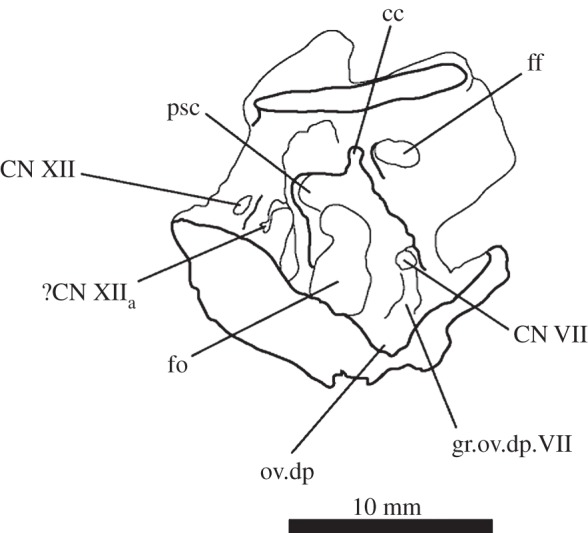
Line drawing of figure 3*b*. Braincase of SAM-PK-7696 in left medial view. For abbreviations, see table 1.

Anterior to the occipital condyle the basioccipital expands laterally to form the basioccipital contribution to the basal tubera (figures 7*b* and 8*b*). A low, rounded ridge extends obliquely from the occipital condyle to about half the distance to the ventrolateral extreme of the contribution on each side, separating a more horizontally orientated ventral surface of the basioccipital from a more vertically orientated dorsal surface (figure 7*b*, rd). In UMCZ T.692, the dorsal parts of the expanded part of the basioccipital contribution on each side appear to be missing. This ridge seems to be the posterior counterpart of the concave articular surface (for the parabasisphenoid) that is located on the anterior face of the contribution of the basioccipital to the basal tuber, as seen in *Prolacerta* [46].

**Figure 5.**
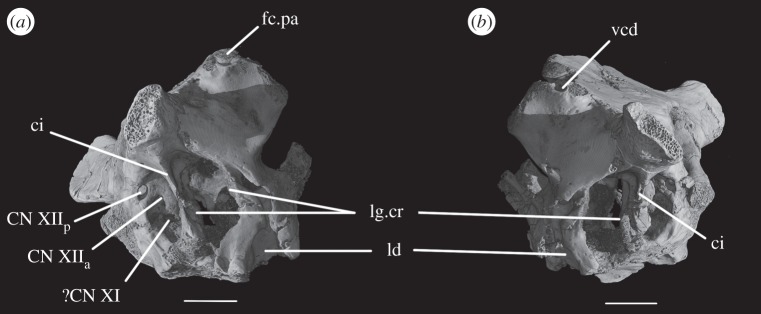
CT reconstructions of the braincase of SAM-PK-7696 (*a*) in right posterolateral and slightly ventral view and (*b*) in left posterolateral and slightly dorsal view. For abbreviations, see table 1.

**Figure 6.**
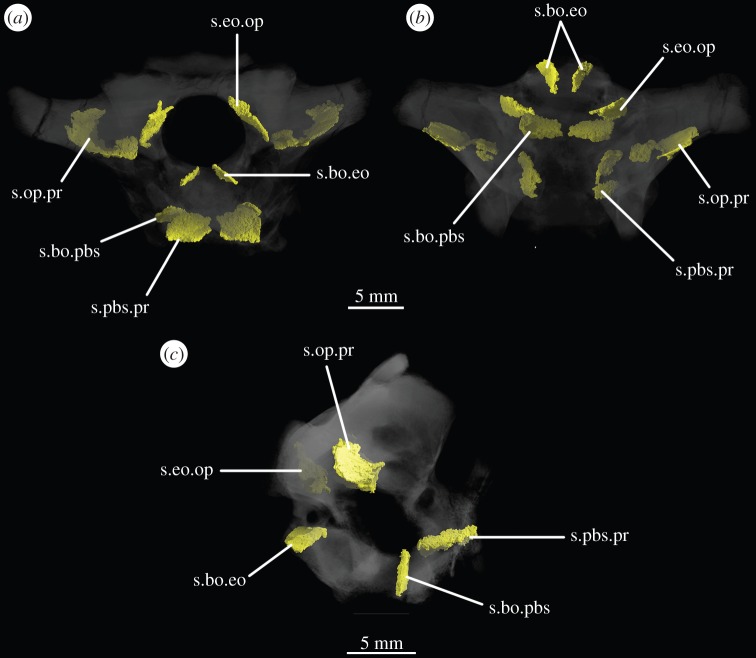
CT reconstructions of the braincase of SAM-PK-7696 showing sutures in (*a*) anterior, (*b*) ventral and (*c*) right lateral views. Bones of the braincase have been rendered transparent for better visualization of the suture lines. For abbreviations, see table 1.

**Figure 7.**
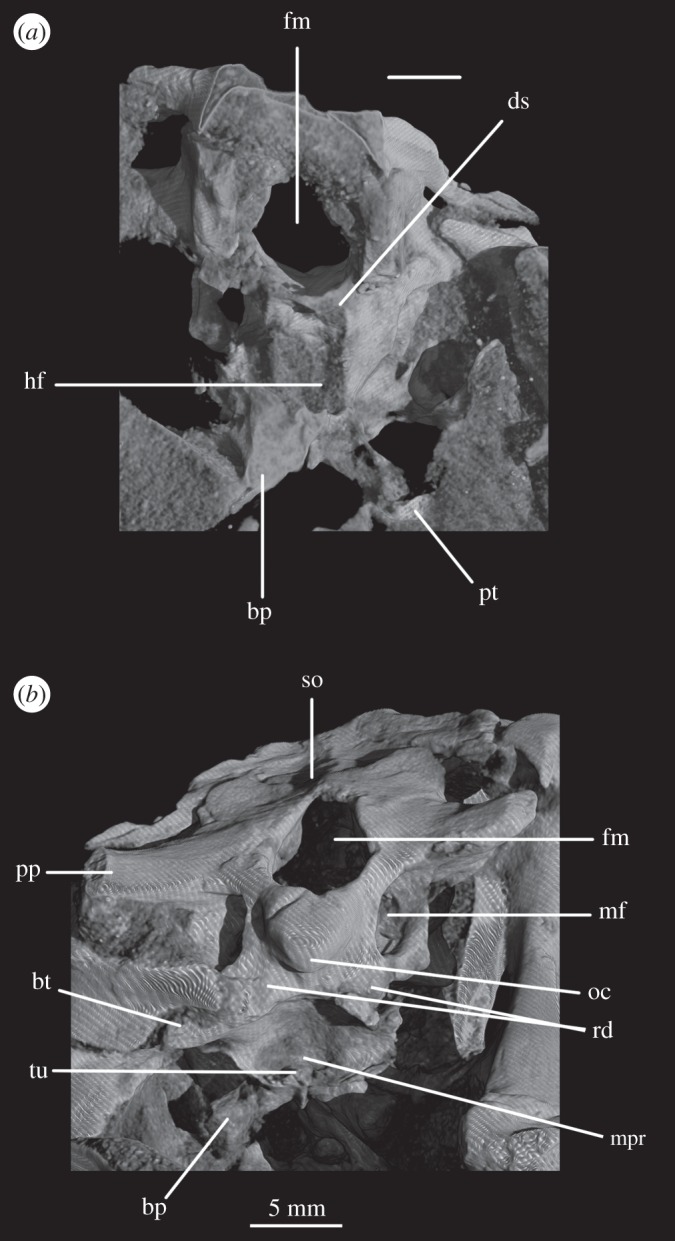
CT reconstructions of braincase of SAM-PK-5867 in (*a*) anterior view in cross section through skull and (*b*) posteroventral view. For abbreviations, see table 1.

The basal tubera are separated in posterior view, but are connected to each other by a low ridge (figures 1*d* and 11*a*, rd) which formed the posterior margin of the basioccipital–basisphenoid fossa [51]. This fossa forms the posterior part of the ventral median pharyngeal recess (*sensu* Witmer [52]; figure 7*b*, mpr); the posterior surface of the parabasisphenoid lacks the ‘intertuberal plate’ that separates the basioccipital–basisphenoid fossa from the rest of the median pharyngeal recess in some other Triassic archosauriforms (e.g. [51]).

The basioccipital also forms the floor of the metotic foramen. The suture between basioccipital and parabasisphenoid extends in a gently meandering line transversely across the braincase, ending laterally close to the posteroventral corner of the fenestra ovalis (figure 3*d*). Thus, the basioccipital contributes to the posterior portion of the floor of the fenestra ovalis; in lateral view, the suture line extends straight ventrally (figure 6*c*).

The lateral margin of the basioccipital dorsal to the basal tuber forms the posterior margin of the ‘unossified gap’ of Gower & Weber [42] (figures 1*e* and 2*e*, ug) also bounded by the ventral ramus of the opisthotic and the parabasisphenoid; the gap is well preserved as an open channel on the right-hand side of SAM-PK-7696 and in SAM-PK-5867 (discussed later; figure 9*a,b*, ug).

**Figure 8.**
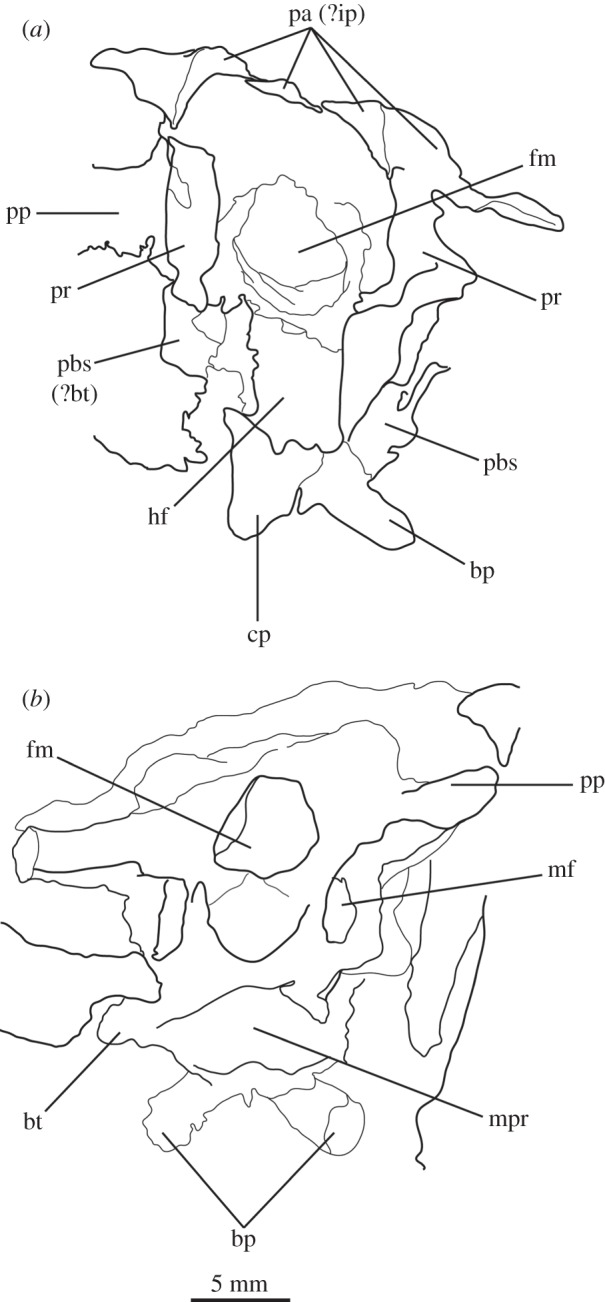
Line drawings of figure 7. Braincase of SAM-PK-5867 in (*a*) anterior view and (*b*) posteroventral view. For abbreviations, see table 1.

### 4.2. Parabasisphenoid

The parabasisphenoid forms the ventral part of the braincase anterior to the basioccipital, ventral to the prootics. The basal tubera are displaced dorsally in comparison to the basipterygoid processes (figure 14*b*), and the part of the parabasisphenoid between them can thus be described as vertically rather than horizontally aligned (following Gower & Sennikov [51]). The basipterygoid processes are well preserved in SAM-PK-K6047A (figure 12*b,c,e,f*), in SAM-PK-5867 (figures 7*b* and 11*a*) and in UMCZ T.692 (figure 14*a,b,e*). They are slightly anteroposteriorly elongated ovals in ventral view, and anterodorsally–posteroventrally elongated ovals in lateral view. The distal tips of the basipterygoid processes are ventrolaterally and slightly posteriorly directed.

**Figure 9.**
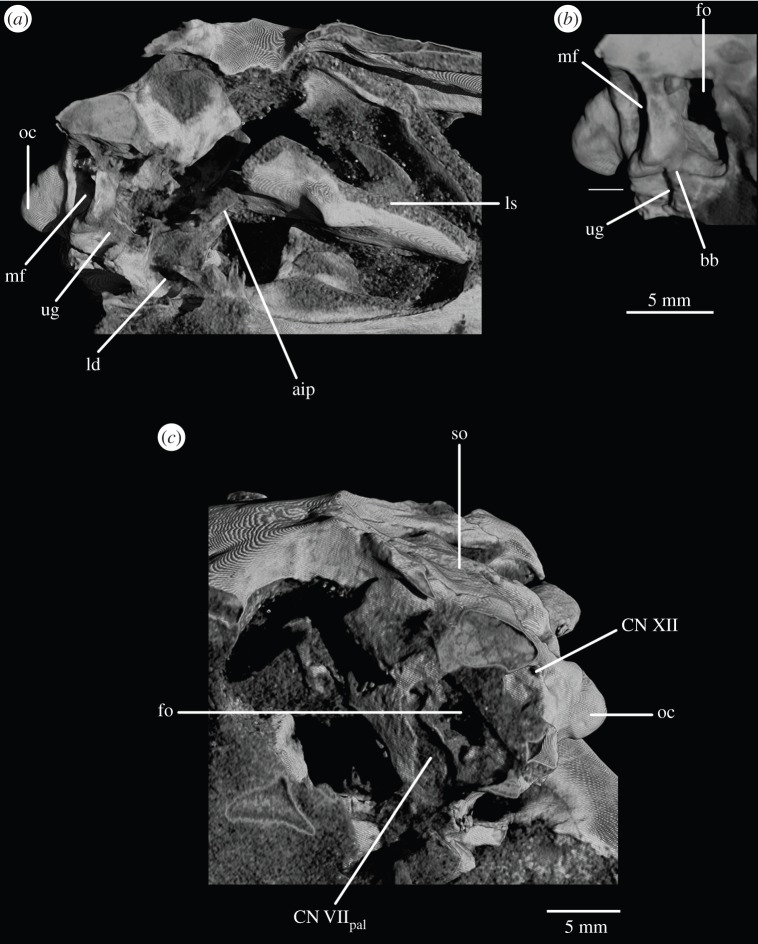
CT reconstructions of braincase of SAM-PK-5867 in (*a*) right lateral view (cross section through skull), (*b*) right lateral view showing the ‘bridge of bone’ (basisphenoid–opisthotic contact) and (*c*) left lateral view (cross section through skull). For abbreviations, see table 1.

**Figure 10.**
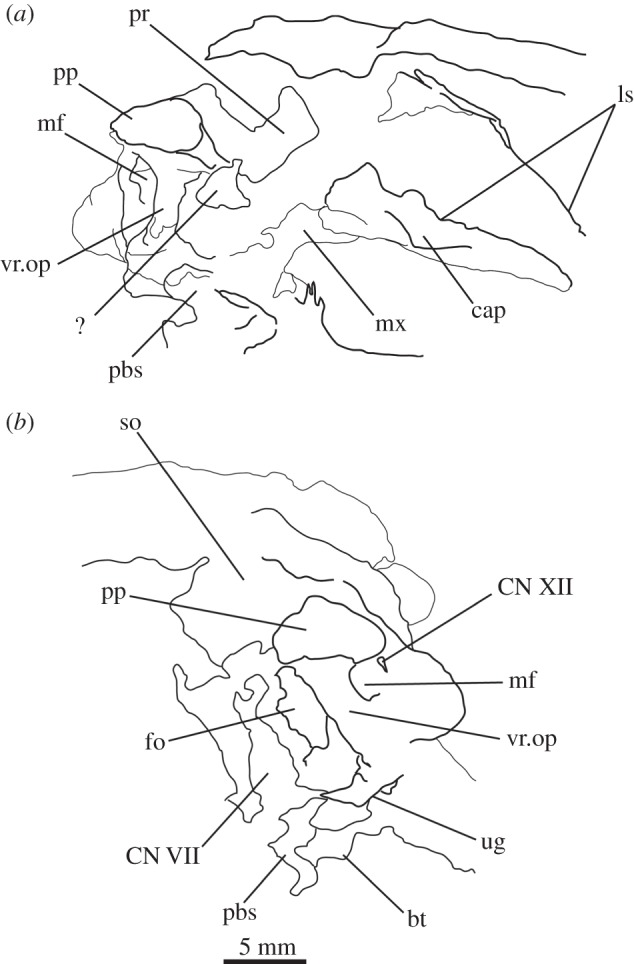
Line drawings of figure 9*a*,*c*. Braincase of SAM-PK-5867 in (*a*) right lateral view and (*b*) left lateral view. For abbreviations, see table 1.

The ventral surface of the parabasisphenoid forms the anterior two-thirds of the median pharyngeal recess (figures 1*d* and 7*b*, mpr) between the basal tubera and the basipterygoid processes. The recess bears no foramina. The suture with the basioccipital extends across the recess in a gently meandering line which is slightly anteriorly convexly curved in overall trajectory in ventral view (figures 1*d*, 3*d* and 7*b*). The anterior bases of the basal tubera are connected to each other by rounded lips of bone that meet in the midline, forming the anterior border of the median pharyngeal recess. They join with a median ridge extending from the ventral surface of the cultriform process, and together form a tubercle which projects posteriorly under the anterior part of the median pharyngeal recess (figure 7*b*).

CT data show that the suture between parabasisphenoid and prootic extends obliquely from posterolaterally to anteromedially in dorsal view (figure 6*b*, s.pbs.pr). However, the parabasisphenoid bears an ascending process posteriorly that conceals part of the lateral surface of the prootic and which forms the anteroventral border of the fenestra ovalis (figure 5*a*). Thus, in lateral view, the contact between prootic and parabasisphenoid can be described in two parts: the first, more posterior part, is anteroventrally inclined and extends from the fenestra ovalis to the groove for CN VII; the second, more anterior part, is anterodorsally inclined and starts anterior to the ‘lateral depression’ of the parabasisphenoid and the groove for CN VII (figure 5*a*). This interpretation of the relationships between these two bones differs from that of previous authors and is discussed in a later section.

The basal tubera are mostly lost in SAM-PK-5867 and completely lost in SAM-PK-7696. However, based on what remains in those two specimens and on UMZC T.692 and SAM-PK-6047A, the parabasisphenoid contribution to the basal tubera extends posteroventrally and laterally from near the anteroventral margin of the fenestra ovalis (figures 12*f* and 14*b,d,e*). On the right-hand side of SAM-PK-7696, the lateral surface of the parabasisphenoid contribution to the basal tuber bears a deep, posteroventrally open sulcus—the semilunar depression of Gower & Weber [42] and Evans [53] (figures 1*e* and 2*e*, sd). This cannot have been an articulation for the ventral ramus of the opisthotic (as suggested by Evans [53]), as the braincase is articulated and the ventral ramus of the opisthotic instead ends more posteriorly, close to the basioccipital contribution to the basal tuber, and connected to the parabasisphenoid laterally by a thin strip of bone. Posterior to the semilunar depression, and anterior to the distal end of the ventral ramus of the opisthotic, is the ‘unossified gap’ of Gower & Weber [42] (figures 1*e* and 2*e*, ug).

Anterior to the anterodorsal extremity of the basal tuber, the lateral surface of the parabasisphenoid is deeply concave (the ‘lateral depression’ of Gower & Weber [42]; figure 2*e*, ld). This concavity is confluent with the groove for the palatine branch of CN VII (figure 2*e*, CN VII_pal_), which extends down the lateral surface of the prootic and would have continued down the anterolateral surface of the basipterygoid process as an osseous groove, as in other reptilians (e.g. *Captorhinus* [54]; *Ctenosaura pectinata* [55]; *Dysalotosaurus lettowvorbecki*, [56]), but is not observable due to preservation.

In lateral view, the posterior third of the braincase floor is subhorizontal, though convex (figure 3*b*). More anteriorly, the floor slopes ventrally (figure 3*b*), and a low median ridge (figure 3*d*, md.rd) divides this sloping section into left and right halves, both of which are gently concave. The anterior third of the floor shows two large, oval depressions (figure 3*b*,*d*, ov.dp) with their longer axes extending posterolaterally–anteromedially. These depressions are a little deeper anteriorly than posteriorly, and they are separated by a thick, dorsally flat strip of the braincase floor, which may have connected to the ridge seen more posteriorly on the braincase floor (this cannot be ascertained because of damage to the braincase floor in SAM-PK-7696).

**Figure 11.**
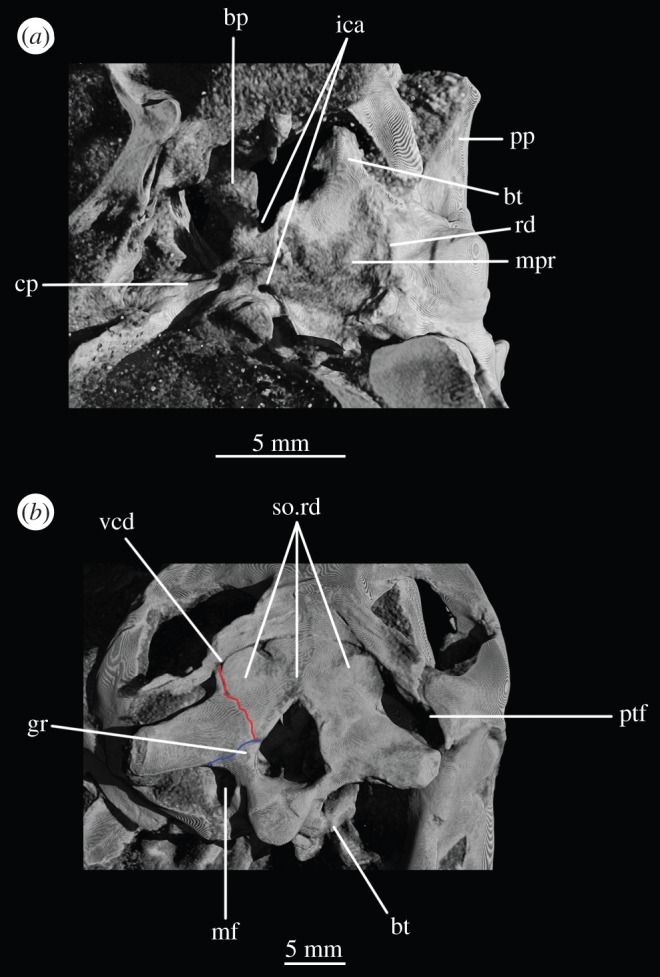
CT reconstructions of braincase of SAM-PK-5867 in (*a*) ventral and (*b*) posterior views. In (*b*), red line indicates the suture between supraoccipital and opisthotic and blue line indicates the suture between opisthotic and exoccipital. For abbreviations, see table 1.

**Figure 12.**
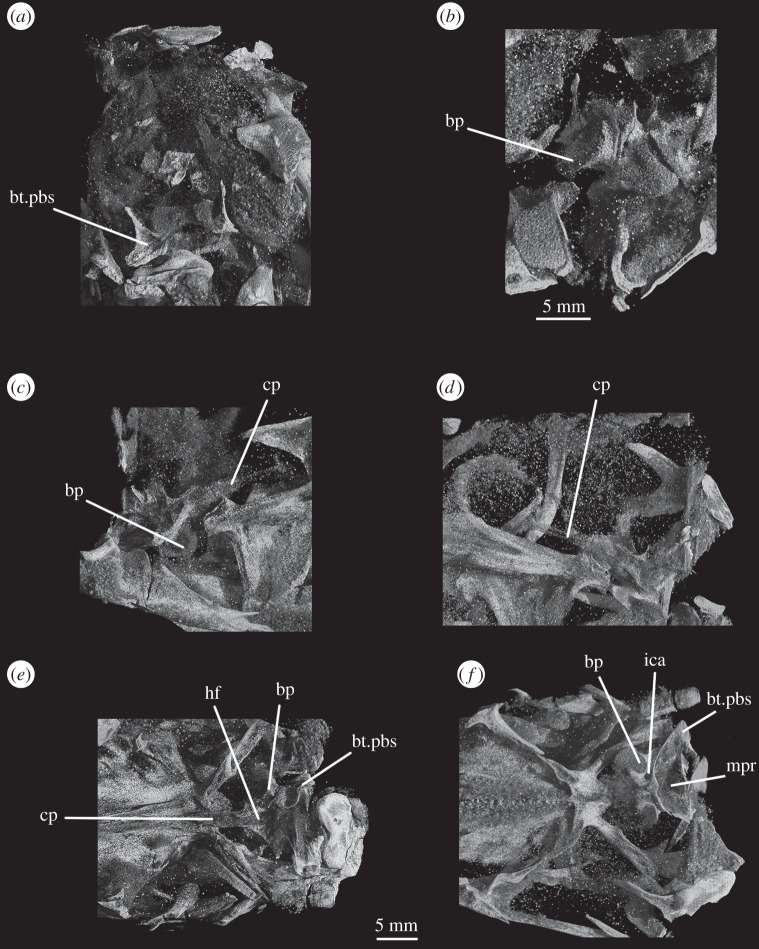
CT reconstructions of braincase of SAM-PK-6047A in (*a*) posterior, (*b*) anterior, (*c*) right lateral, (*d*) left lateral, (*e*) dorsal and (*f*) ventral views. For abbreviations, see table 1.

The ventral surface of the parabasiphenoid between the basipterygoid processes is very gently concave, with a pronounced median ridge extending from the anterior margin of the median pharyngeal recess to the base of the cultriform process (=rostrum) of the parabasisphenoid (figure 11*a*). This ventral surface bears, on each side, a foramen for the internal carotid artery (figure 11*a*, ica), placed at the posteromedial base of the basipterygoid process, immediately anterior to the lips of bone connecting the basal tubera (as mentioned earlier). The cultriform process (figures 12*c–e*, cp and 14) is elongated and tapers to a distal point, and its dorsal margin dips slightly ventrally close to its base then rises dorsally again yet further proximally. In cross section, the cultriform process is deeply excavated dorsally, forming a U-shape in anterior view.

In anterior view, the suture between the parabasisphenoid and the prootic extends from ventrolaterally to dorsomedially, through the foramen of CN VI on each side (the margin of which is thus formed half by the parabasisphenoid and half by the prootic), meeting in an apex at the midline close to the dorsal border of the dorsum sellae (figure 2*a*). The posterior wall of the hypophyseal fossa is gently concave transversely and has a central, low ridge extending dorsoventrally. The clinoid processes (figures 1*a* and 2*a*, cp) protrude a small distance medially over the posterior wall of the hypophyseal fossa, concealing the lateral borders of the foramina for CN VI in anterior view.

### 4.3. Exoccipital

The contact between exoccipital and basioccipital is very short (figure 6), confirming the observation of Gower & Weber [42] that the exoccipital is restricted to the pillar between the foramen magnum and the metotic foramen. The lateral surface of this pillar is smooth and shows no lateral ridge (*sensu* Gower [57]; figure 1*e*). The exoccipitals form the dorsolateral corners of the occipital condyle, and in SAM-PK-7696 do not meet at the midline to exclude the basioccipital from the foramen magnum (figures 1*b* and 2*b*). On first inspection, SAM-PK-5867 presents the impression that the exoccipitals did exclude the basioccipital from the foramen magnum (figure 7*b*), but this appears to be due to mediolateral compression of the braincase compounded by a preparation artefact. In UMZC T.692, the left side of the braincase is disarticulated and was not scanned with the main block of the material. The right exoccipital, however, is preserved in contact with the basioccipital (figure 14*b*) and a line of fracture likely represents their contact. In this specimen, the exoccipital extends further medially than in SAM-PK-5867, almost reaching the midline, but does not seem to contact its counterpart, as noted by Gower & Weber [42]. Although the braincase is not laterally compressed, the exoccipital seems to be somewhat displaced medially from its original position, decreasing the distance between left and right elements. In addition, the exoccipital facets on the basioccipital are directed slightly outwards, again indicating lack of contact. We thus conclude that there is a basioccipital contribution to the foramen magnum, but this contribution seems to be smaller in SAM-PK-5867 and UMZC T.692 than in SAM-PK-7696. The basioccipital is missing in SAM-PK-6047A.

The anterior two-thirds of the suture between the exoccipital and opisthotic are identifiable in CT scans of SAM-PK-7696, and in posterior view the suture is slanted dorsomedially–ventrolaterally (figure 6*b*, s.eo.op). Exactly where this suture emerges posteriorly is not entirely clear in any specimen, but based on the part of the suture visible internally in the scans, its path would roughly describe an arch that begins at the base of the paroccipital process and reaches up to the dorsal border of the foramen magnum. On the left side of SAM-PK-7696, there is a meandering line that broadly follows this same arch (figures 1*b*, red line and 2*b*). This line separates a more ventral, smoother and depressed area from a more dorsal, rougher and more convex area and almost certainly represents the exoccipital–opisthotic suture as it follows its expected trajectory, though its continuation cannot be traced internally; a slight groove is visible in SAM-PK-5867 in a similar position to the line in SAM-PK-7696 (figure 11*b*, blue line). In SAM-PK-7696, the posterior surface of the exoccipital is damaged at the point where this probable suture line would contact the border of the foramen magnum, preventing clarity regarding the exact contribution of the exoccipital to the foramen. However, it is probable, based on the suture line within the bone, that the exoccipital formed the lateral rim of the foramen magnum as well as the lateral parts of its dorsal rim. In SAM-PK-7696, the supraoccipital–opisthotic suture cannot be located, but in SAM-PK-5867, the supraoccipital–opisthotic suture line hits the probable exoccipital–opisthotic suture line immediately lateral to the border of the foramen magnum (figure 11*b*, red line), meaning that the opisthotic is excluded from the foramen, and the suture between the supraoccipital and exoccipital consists of a brief point contact at the border of the foramen magnum.

A depressed area (as mentioned earlier) below the exoccipital–opisthotic suture line is clearly present on the posterior surface of the exoccipital in SAM-PK-7696, being especially pronounced on the right-hand side (figure 1*b*). This feature is less apparent in SAM-PK-5867 (figure 11*b*). Whether this area represents a particular functional feature is unclear, but it certainly does not represent any exit foramina, as the CT scans show no traces of internal paths. In SAM-PK-7696, on the right lateral surface of the exoccipital, there are two well-marked foramina for the anterior and posterior branches of CN XII (figure 5*a*, CN XII_a_, CN XII_p_)—the posterior foramen (CNXII_p_) is somewhat larger and more dorsally located. A short distance anterior and ventral to these, there seems to be a third foramen, which, in the CT scans, does not penetrate far into the bone (figure 5*a*, ?CN XI). If a real feature, however, this foramen could represent an independent exit for the accessory nerve (CN XI). On the left side, the foramen for the posterior branch of CN XII is clearly visible, but because the area anterior to it is somewhat damaged, the foramen for the anterior branch of CN XII is located more medially. There is no corresponding foramen to the third foramen seen on the right-hand side. In SAM-PK-5867, only one foramen is visible in this area of the exoccipital, corresponding to the exit of the posterior branch of CN XII (CN XII_p_).

### 4.4. Opisthotic

The opisthotic forms most of the paroccipital processes and the lateral wall of the braincase between the fenestra ovalis and the metotic foramen. Contrary to Cruickshank [45], the opisthotic does not participate in the borders of the foramen magnum. The contact between opisthotic and prootic is broad (*sensu* Nesbitt [28], state 0 of character 105), being dorsoventrally extended and reaching up to half the length of the paroccipital process (figure 6). However, the prootic contribution to the paroccipital process is restricted to a sheet of bone covering the anterior surface of the process, the bulk of the process being formed by the opisthotic (figure 1*a,e*).

The paroccipital processes protrude posterolaterally and dorsally (figures 1, 7, 11). They are oval in cross section and the shaft is gently twisted along its length, so that the dorsal surface twists to face more posterodorsally at the distal end. The distal tips are gently rounded and separated from the main shaft of the processes by a slight constriction. The distalmost tip of the right process is missing in SAM-PK-7696. The paroccipital processes are excavated postero- and anteroventrally by the dorsal borders of the metotic foramen and the fenestra ovalis (recessus stapedialis), respectively. These excavations are separated by a ridge (corresponding to the crista interfenestralis of Oelrich [55]) ascending from the ventral ramus of the opisthotic and ending approximately half way along the paroccipital process shaft (figure 5*a*, ci).

The ventral ramus of the opisthotic (figures 1*b*,*f* and 2*b*,*f*, vr.op) descends ventrally from the base of the paroccipital process and bends gently posteriorly. It separates the fenestra ovalis anteriorly from the metotic foramen posteriorly. In posterior view, the ventral ramus of the opisthotic is clearly visible, with its lateral margin offset laterally from that of the exoccipital by a distance roughly equal to the width of the exoccipital (figures 1*b* and 2*b*, vr.op). In posterior view, the lateral margin of the ventral ramus of the opisthotic is laterally concave and extends from ventromedially to dorsolaterally. The distal end of the ventral ramus of the opisthotic is roughly level with the dorsoventral midpoint of the occipital condyle. In transverse cross section, the long axis of the ramus is anteromedially to posterolaterally directed; the perilymphatic duct would thus have extended anterolaterally to posteromedially (discussed later). On the anterior surface of the left ventral ramus of SAM-PK-7696, the well-marked lagenar crest protrudes anteriorly (figure 5*a,b*, lg.cr), separating the vestibular region dorsally from the cochlear region ventrally. This structure is missing on the right side. The distal end of the ventral ramus of the opisthotic is expanded laterally, anteriorly and posteriorly compared with the rest of the shaft, but this expansion does not compare with that seen in some other stem archosaur taxa (e.g. *Garjainia prima* [51]).

On the ventral border of the right fenestra ovalis in SAM-PK-7696, there is a thin sagittal bony contact between the ventral ramus of the opisthotic and the posterior region of the parabasisphenoid (figure 2*e*, bb). This bony contact forms the lateral limit of a small foramen (figure 2*e*, ug), the medial edge of which is delimited by the lateral surfaces of the basioccipital and the parabasisphenoid forming the braincase floor. The ventrolaterally open area ventral to this foramen was identified by Cruickshank [45] as the lagenar recess, whereas Gower & Weber [42] identified it as an ‘unossified gap’. This area was probably covered by cartilage, with the tip of the lagena projecting through the foramen (discussed later).

On the dorsal part of the medial wall of the opisthotic, there are two confluent, medially open depressions (figure 3*a,b*, cc, psc). One is smaller and positioned more anterodorsally than the other. The first corresponds to the common, dorsal openings of the anterior and posterior semicircular canals, termed common crus. The second, posteroventral one corresponds to the posterior ampulla, from which the posterior semicircular canal leaves the vestibule. On the right-hand side of SAM-PK-7696, the ventral ramus of the opisthotic has a large, rounded notch occupying all the region ventral to the confluence of these two depressions (figures 3*c* and 16*e,f*, pf). This notch marks the border between the otic capsule and the occipital region, through which passed the perilymphatic duct. The notch is, however, too large to be considered only the lateral border of the perilymphatic foramen, and it may have housed other structures such as part of the perilymphatic sac (see inner ear and discussion sections below).

### 4.5. Prootic

The prootic forms the lateral wall of the braincase posterior to the laterosphenoid and anterior to the fenestra ovalis (figures 1*e,f* and 2*e,f*, pr). Posterolaterally, the prootic extends onto the anterior surface of the paroccipital process in a laterally tapering sheet that reaches to just under half way along the paroccipital process (figure 1*a,e,f*). Anteriorly, the prootic contacts the laterosphenoid, and forms the dorsal, posterior and ventral margins of the large foramen for the trigeminal nerve (CN V), with the laterosphenoid forming the anterior margin. The CN V foramen (figure 1*f*, CN V) is oval, with its long axis extending posteroventrally to anterodorsally. The anterodorsal extremity of the prootic forms the ventral floor of a recess (figures 1*a,c,e,f*, 3*a* and 5*b*, vcd) which was roofed by the supraoccipital dorsomedially and, when in articulation, by the parietal dorsolaterally. The laterosphenoid may have formed the anterior wall of this channel, though it is disarticulated in all specimens. The smooth and rounded nature of this recess, and the relationships of the cited elements as seen in other taxa suggest that it was not simply an articulation for the parietal and/or laterosphenoid, but a channel for the vena capitis dorsalis [56,58–60], which would have connected the braincase cavity with the temporal region. In the CT scans of SAM-PK-5867, whether such a channel is present is difficult to assess, but there does appear to be a rounded opening which may represent its lateral extreme (figure 11*b*). A corresponding structure identified as a venous sinus is found on the supraoccipital of *Osmolskina czatkowicensis* [61]. Immediately posterior to the lateral part of this recess is a small posterolaterally directed depression with marked borders which appears to have been a facet for the parietal (figure 5*a*, fc.pa).

Posteroventral to the foramen for CN V, the prootic is slightly depressed, indicating the position of a Gasserian ganglion external to the brain cavity (figures 1*e*,*f* and 2*e*,*f*, gr.ga). A sharp crest (here referred to as crest 1, to avoid terminological confusion) extends ventrally down from the paroccipital process (figure 2*e*, cr1), forming the anterior margin of the stapedial groove and then, ventral to the dorsal margin of the fenestra ovalis, the anterior margin of the groove for the hyomandibular branch of CN VII (CN VII_hym_, figure 1*e*). A lower crest (crest 2; figure 2*e*, cr2) originates from this crest at the dorsal margin of the fenestra ovalis, and forms the posterior margin of the dorsalmost part of the groove for CN VII_hym_ and then the anterior margin of the fenestra ovalis. Crest 1 becomes much lower and arcs anteriorly then posteriorly again just below the exit for CN VII, before descending directly ventrally and approaching the posterior wall of the groove for the palatal branch of CN VII (CN VII_pal_). Further ventrally still, the wall of the groove for CN VII_pal_ and crest 1 diverge once again below to form the posterior and anterior margins of the lateral depression, respectively. A bulging ridge marking the path of the lateral semicircular canal follows the line of crest 1 dorsal to the foramen for CN VII, but is inset anterodorsally from the crest. Once it reaches a point level with the foramen for CN VII, this ridge curves sharply anterodorsally, bordering the dorsal margin of the depression for the Gasserian ganglion (figure 2*e*). In lateral view, the posterior surface of the prootic forming the anterior border of the fenestra ovalis of SAM-PK-7696 bears a lagenar crest (figure 5*a,b*, lg.cr), a smoothly rounded protuberance that marks the division of the vestibular and cochlear regions of the inner ear.

The medial wall of the prootic possesses a large, round and very deeply marked fossa immediately dorsal and a short distance posterior to the foramen for CN V—the floccular fossa (= fossa subarcuata, auricular fossa; figures 1*a* and 3*a,b*, af). The posterior wall of the fossa has a marked, deeper subregion that seems to enter the bone but does not lead off within it. Ventral to the floccular fossa, the left foramen for CN V is separated from the inner ear by an elevated and rounded ridge (figures 3*b*, rd and 4). Posterior to this ridge, close to its base, there seems to be a dorsoventral sulcus, perhaps leading dorsally to the foramen for CN VII or ventrally to the brain cavity (figure 3*b*, su). The foramen for CN VII is connected to the oval depressions (as mentioned earlier) on the anterior third of the braincase floor by a groove (figure 4, gr.ov.dp.VII).

**Figure 13.**
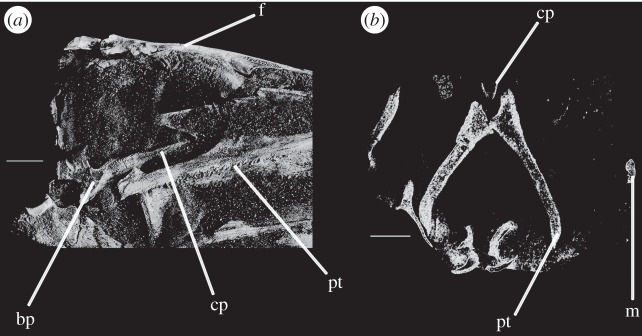
Details of the cultriform process of SAM-PK-6047A in (*a*) right lateral and (*b*) transverse views. For abbreviations, see table 1.

Ventral to the foramen for the trigeminal nerve, the prootic projects as a thin process—the anterior inferior process (figure 2*e*, aip). The left and right processes are connected by a sheet of bone, the dorsum sellae (figure 2*a*, ds). The dorsum sellae is bordered laterally by ridges, which connect ventrally to the protruded margins of the clinoid processes (figure 2*a*, rd; see parabasisphenoid). The middle third of the dorsal margin of the dorsum sellae dips ventrally to the midline, thus forming a V-shape in anterior view (figure 1*a*). The dorsum sellae forms the dorsal rim of the foramen of the abducens nerve (figure 2*a*, CN VI), and is directed anteroventrally rather than anteriorly, unlike, for example, in *Erythorosuchus africanus* [62]. On the dorsal part of the lateral surface of the anterior inferior process, immediately ventral to the trigeminal opening, is a low, very slight anteroposteriorly extending narrow ridge (figure 2*f*, rd); it is less pronounced than in *Dorosuchus* (PIN 1579/62) and is perhaps better described as a thickening of the bone along the margin of the trigeminal foramen rather than a true ridge.

### 4.6. Supraoccipital

The supraoccipital is a broad, flat element that forms the posterodorsal part of the roof of the braincase and forms the medial third of the dorsal border of the foramen magnum (figures 1*b,c*, 2, 7*b*, 9*c*, so and 11*b*). The supraoccipital partially housed the common crus, the posterior portion of the anterior semicircular canal and the anterior part of the posterior semicircular canal.

Laterally and posterolaterally, the supraoccipital contacts the opisthotics. The entire line of this suture is unclear in SAM-PK-7696. In SAM-PK-5867, however, in dorsal view, this suture extends in a laterally concave arc from the lateral margin of the contact between the exoccipital and the supraoccipital at the lateral margin of the foramen magnum to the anteromedial margin of the paroccipital process (figure 11*b*). Anterior to the end of this suture, the supraoccipital contacts the prootic along the dorsolateral extreme of the braincase (figures 2*e,f* and 11*b*). The suture line could not be identified in SAM-PK-7696, but in SAM-PK-5867 it describes a gentle, medially concave arc from the lateralmost point of the suture between supraoccipital and opisthotic to the anterior border of the supraoccipital, at the dorsomedial corner of the recess of the vena capitis dorsalis (figures 3*a*, 11*b*, vcd). The lateral part of the dorsal margin of the supraoccipital thus forms the medial and posterior margins of the recess (see ‘Prootic’ section), which is anteriorly and dorsally open; immediately medial to this, the supraoccipital also roofs the medial section of this hollow.

In posterior view (figure 11*b*), the dorsal margin of the supraoccipital is raised into a convexity at the midline and into another, smaller convexity laterally on either side. Gentle ridges or raised strips extend back from each of these convexities (figure 11*b*, so.rd), with those extending from the lateral convexities moving towards the midline posteriorly (figure 11*b*); all three of these ridges disappear around half way to the border of the foramen magnum.

### 4.7. Laterosphenoid

In SAM-PK-5867, the braincase wall anterior to the prootic is fully ossified as the laterosphenoid, although disarticulated from the remainder of the braincase (figure 15), indicating that it may not have been firmly sutured in life. In SAM-PK-5867, the laterosphenoid is displaced anteriorly and (probably associated with the lateral compression of the specimen seen on its left side) greatly tilted to the right in relation to the occipital area of the skull, so it is visible in dorsal view through the right orbit (figures 15 and 17*a*). A single disarticulated bone identified as a right laterosphenoid has been described from SAM-PK-7696 [63]. Given its fragmentary nature and lack of convincingly diagnostic features, we find that whether this is indeed a laterosphenoid (and/or the same ossification of the anterior braincase wall as preserved in SAM-PK-5867) is difficult to assess. Fragments of bone were attributed to the laterosphenoid in UMZC T.692 [42,63] and this attribution is confirmed by CT scans, but their poor preservation prevents morphological information being obtained from them. The laterosphenoid of SAM-PK-6047A is missing entirely. The presence of a laterosphenoid was noted by Clark *et al*. [63] for SAM-PK-5867, but that specimen was not fully prepared at that time to allow a more complete description. Although we agree with these authors on the identification of this structure as a laterosphenoid, we note some differences to the laterosphenoid of extant crocodilians, potentially indicating the presence of more elements (fused together) in *Euparkeria* than the ‘true’ laterosphenoid (discussed later). Irrespective of homology, the laterosphenoid of *Euparkeria* will be described here as a single element. The description is based on the right side of the laterosphenoid of SAM-PK-5867, unless stated otherwise, as this is more clearly visible both visually and in the CT scans.

**Figure 14.**
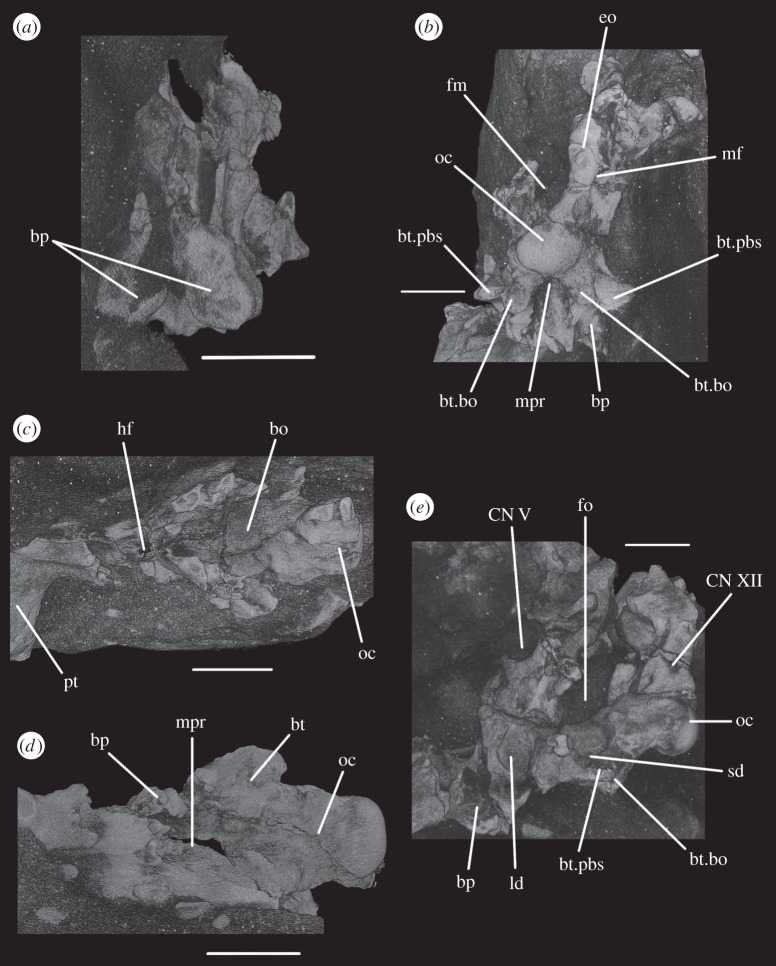
CT reconstructions of braincase of UMZC T.692 in (*a*) anterior, (*b*) posterior, (*c*) dorsal (in cross section to expose braincase floor), (*d*) ventral and (*e*) left lateral views. For abbreviations, see table 1.

**Figure 15.**
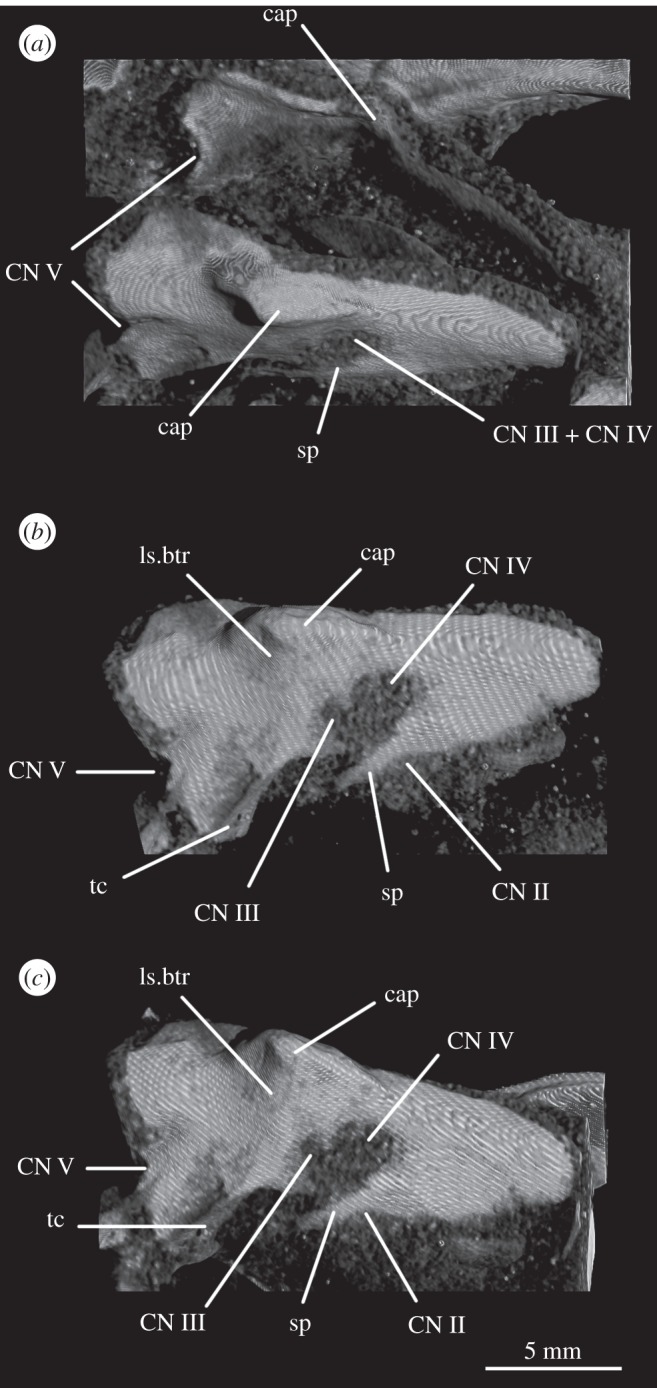
CT reconstructions of laterosphenoid(s) of SAM-PK-5867 in (*a*) right dorsolateral, (*b*) right lateral and (*c*) right ventrolateral views. For abbreviations, see table 1.

The laterosphenoid of *Euparkeria* is fundamentally similar to that of *Proterosuchus alexanderi* (NMQR 1484), but less anteroposteriorly elongated. The laterosphenoid of *Euparkeria* does not reach further anteriorly than half the length of the orbit, while that of *Proterosuchus alexanderi* does. The anterior part of the laterosphenoid is also more ventrally extended (figure 15*b,c*). In dorsal view, the posterior part of the dorsal rim extends straight anteriorly, then bends sharply anterolaterally to form the posterior part of the capitate process, and then curves smoothly anteromedially again (figure 15*a*). In lateral view (figure 15*b*), the dorsal half of the posterior margin of the laterosphenoid is convexly rounded. Although it shows a subtle projection, it has no posteriorly extending process as developed as that seen in *Proterosuchus alexanderi* [63]. The dorsal half of the posterior margin of the laterosphenoid is separated from the ventral half by a notch, which forms the anterior rim of the foramen for CN V (figure 15*b*, CN V). The border of this notch is depressed laterally, as seen in the prootic, marking the position of the Gasserian ganglion. The ventral half of the posterior margin of the laterosphenoid is still in articulation with the anterior inferior process of the prootic (figures 9*a* and 15*b,c*); the laterosphenoid does not extend below the articulation with the prootic, and would not have contacted the parabasisphenoid. The lateral surface of the posterior part of laterosphenoid is smooth and delimited anteriorly by two rounded crests: a more dorsal crest, the laterosphenoid buttress (=cotylar crest of Clark *et al*. [63]; figure 15*b,c*, ls.btr), and a more ventral crest, the tensor crest (*sensu* Holliday & Witmer [64]; figure 15*b,c*, tc).

The laterosphenoid buttress curves first anteroventrally and then posteroventrally from the capitate process (figure 15, cp) to form an anteriorly convex outline with the tensor crest. Both structures, however, do not contact each other, leaving a space between them flush with the lateral surface of the laterosphenoid, at about its midheight. The capitate process (= postorbital process of Holliday & Witmer [64]:718; figure 15, cp) is very robust in comparison to the rest of the laterosphenoid, and protrudes from the main body of the laterosphenoid laterally. The tensor crest (figure 15*b,c*, tc) of *Euparkeria* is very well marked compared with that of *Proterosuchus alexanderi*, where it appears to be absent [63]. Anterior to the tensor crest, the laterosphenoid extends as a medially directed process, the dorsoventral extension of which is about half that of the tensor crest.

Anterior to the buttress and to the tensor crest, there is a large opening. The posterior margin of this opening bears one sharp, distinct anterior projection, just ventral to the end of the laterosphenoid buttress. On the right-hand side, this projection does not appear to reach the anterior margin of the opening, but on the left-hand side, it reaches the anterior margin to form a small foramen dorsal to it. Although visible in the specimen, the complete bar forming the foramen of the left-hand side could not be segmented out in the three-dimensional model because, despite having a different coloration from the matrix, the densities are not easily distinguishable. This bar would represent the separation between the foramina of CN III ventrally from CN IV dorsally.

The anteroventral borders of the CN III and IV foramina are formed by the slender process. Compared with *Proterosuchus alexanderi* (NMQR 1484; Clark *et al*. [63]), the slender process of *Euparkeria* (figure 15, sp) is longer and more posteriorly directed. The anterodorsal border of the slender process seems to be slightly notched, probably corresponding to the point of exit of CN II (figure 15*b,c*, CN II). No ventral crest that would correspond to that identified in *Proterosuchus alexanderi* [63] is visible. The lateral surface of the anteriormost region of the laterosphenoid is smooth and gently concave. The laterosphenoid tapers dorsoventrally at its anteriormost end (figure 15*b,c*), but, in relation to the width of the slender process, it does not extend as far anteriorly as that in *Proterosuchus alexanderi* [63].

### 4.8. Inner ear

**Figure 16.**
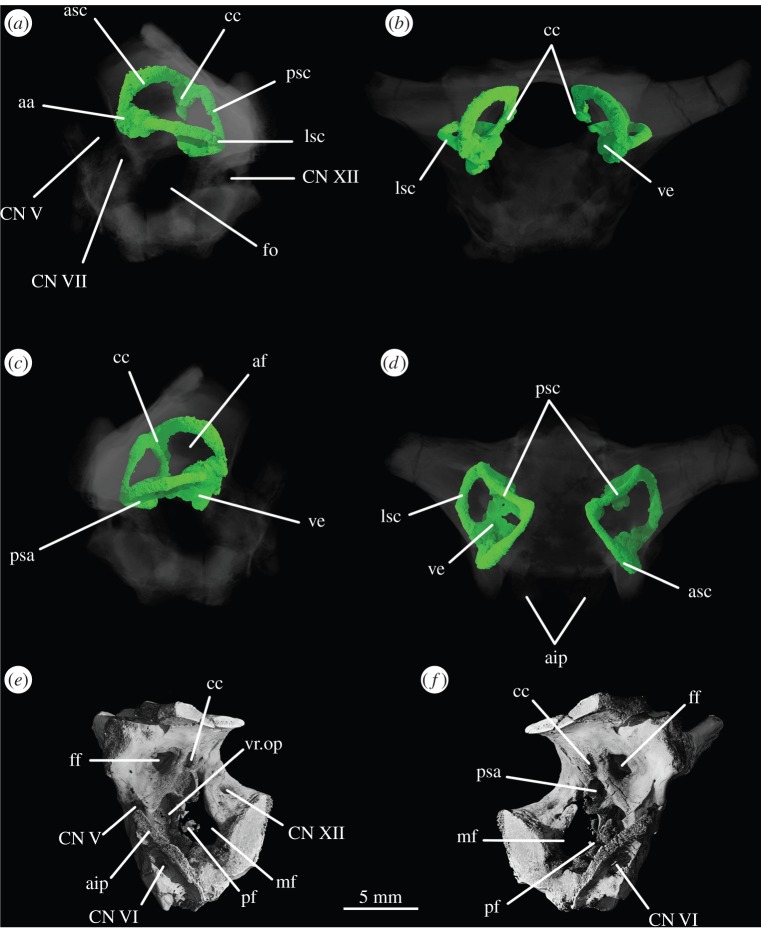
CT reconstructions of braincase of SAM-PK-7696 showing structures of inner ear in (*a*) left lateral, (*b*) anterior, (*c*) right lateral and (*d*) dorsal views, and showing (*e*) right medial wall in anteromedial view and (*f*) left medial wall in anteromedial view. In (*a*–*d*), bones of the braincase have been rendered transparent for better visualization of the suture lines. For abbreviations, see table 1.

The inner ear (figure 16) is very well preserved and when the overall size of the braincase is taken into account, it is much enlarged (82.44 mm^3^; table 2) when compared with that of *Youngina* (62.69 mm^3^ [50]; the only non-saurian diapsid for which the inner ear is segmented). The fenestra ovalis (figure 16*a*, fo) is well defined and dorsoventrally elongate when compared with that of *Prolacerta* [46, fig. 1] and laterally it is formed mostly by the prootic anteriorly and the opisthotic posteriorly, with participation of the parabasisphenoid ventrally and anteroventrally (figure 6*c*). The basioccipital also contributes to the posteroventral part of the medial margin (figure 4*c*). The lagenar crests (figure 5*a,b*, lg.cr) are situated on the anterior and posterior borders of the fenestra ovalis, at about its midheight. The lagenar crests mark the dorsalmost limit of the lagenar recess and separate the vestibular and cochlear regions of the inner ear (figure 5*a,b*, lg.cr). The anterior lagenar crest is low and rounded, while the posterior one is more prominent and thinner. The ventralmost tip of the lagenar recess appears to lie in the ‘unossified gap’ (*sensu* Gower & Weber [42]), with the cochlea having passed medial to the bony bar connecting the ventral ramus of the opisthotic and the posterodorsal region of the parabasisphenoid, and lateral to the braincase floor (figure 3*d,f*). The region connecting the otic capsule and the occipital region in *Euparkeria* is marked by a rounded notch on the medial side of the ventral ramus of the opisthotic (figures 3 and 16*e,f*, pf). The perilymphatic duct passed through this notch, likely through its narrowest part. This part is located more laterally in comparison to that of *Sphenodon* (ROM R9298) and the duct ran in a more anteroposterior direction in *Euparkeria* as opposed to anterolateral to posteromedial in *Sphenodon* (as discussed in later sections). The notch as a whole is too large to have housed the perilymphatic duct alone. Thus, the perilymphatic foramen was only partially laterally surrounded by bone (see ‘Opisthotic’ section).

**Table 2.** Measurements of the inner ear of *Euparkeria*. The measurements of the vestibule used to compare the volume with *Youngina* in the text were taken from the right side because that is the only information available for *Youngina*. The measurements for the semicircular canals are taken from one ampulla to the other, or to the common crus, thus corresponding to the circumference of the canals at their longest point.

	right (mm)	left (mm)
vestibule, height	4.34	4.31
vestibule, length	6.29	5.93
vestibule, width	3.02	2.85
asc	6.64	6.88
psc	5.83	5.89
lsc	5.32	6.01

The semicircular canals (figure 15*a–d*) are of roughly the same length as one another. The posteroventral part of the vestibule is not enclosed by bone, in part because of the elongated fenestra ovalis, and medially the internal auditory meatus is largely unossified. The anterior semicircular canal exits the anterior ampulla anteriorly and slightly laterally (figure 16, asc), extending immediately dorsally and posteriorly, and then medially, around the floccular fossa, entering the common crus anterodorsally. Only the dorsalmost part of the common crus is surrounded by bone (figure 16*e,f*, cc), but its ventral connection to the utriculus is marked by a groove on the right medial wall of the braincase (figure 3*a*, gr.ut), allowing its course to be reconstructed. The anterior and posterior semicircular canals meet at approximately the midlength of the vestibule (figure 16*a*, asc, psc), but the common crus enters the utriculus just anterior to the dorsal rim of the fenestra ovalis, extending ventrally and posteriorly. The posterior semicircular canal leaves the posterior portion of the vestibule dorsolaterally and extends anteromedially into the common crus. In dorsal view (figure 16*d*), the paths of the anterior and posterior semicircular canals describe arches whose concavities face opposite directions. The lateral semicircular canal is a little shorter than the other semicircular canals. It leaves the posterolateral portion of the anterior ampulla, describing a gentle arc in dorsal view, and enters the vestibule laterally (figure 16*d*).

Like the fenestra ovalis, the metotic foramen (figures 1*f*, 7*b* and 16*c--f*, mf) is also dorsoventrally elongated compared with *Youngina* [50, online animated figure] and *Prolacerta* [46, fig. 1]. The lateral border of the metotic foramen is fairly uniform, but the posterior rim of the ventral part of the medial border protrudes, so that the foramen is wider at its medial margin ventrally than dorsally. The ventral portion comprises more than half of the foramen. The metotic foramen is not subdivided into a fenestra pseudorotunda and a vagus foramen and there is also no indication of an independent exit for the glossopharyngeal nerve, thus it is certain that CN X and CN IX exited the braincase through the metotic foramen. The accessory nerve (CN XI) and the vena cephalica posterior could have exited the braincase through the metotic foramen, or, alternatively, through the faint, anteriormost foramen found on the right exocciptal of SAM-PK-7696 (as discussed earlier; figure 5*a*, ?CN XI) and through the foramen magnum, respectively. The irregular medial shape of the metotic foramen potentially indicates the positions of the structures housed by, and exiting through, the foramen: the ventral portion is wide and rounded in posterolateral view, and likely represents the area where the perilymphatic sac sat and bulged into—the area corresponding to the recessus scalae tympani in taxa where the fenestra pseudorotunda is present. The perilymphatic foramen, which connects this area to the lagenar recess, is only ossified laterally, as described earlier. The nerves and the vein would have left dorsal to the perilymphatic sac, where the metotic foramen is narrower medially.

### 4.9. Cranial nerves and some vascular elements

The optic nerve (CN II) would have entered the braincase anteromedially either through a single foramen or through separate foramina for the right and left nerves. The laterosphenoid of *Euparkeria* is fairly well preserved, thus the absence of any medial structure, or indication thereof, seems to indicate that the CN II left the braincase through a single medial foramen (figure 16*b*,*c*, CN II), similar to the situation in *Proterosuchus alexanderi* [63]. The oculomotor (CN III) and trochlear (CN IV) nerves are closely related motor nerves that may have confluent foramina in some archosaur clades [65], with the CN III lying ventral to the CN IV. The nerves in *Euparkeria*, like in *Proterosuchus alexanderi* [63], had separate foramina (figure 16*b*,*c*, CNIII, CNIV), but the ventrolateral border of the foramen for CN III in *Euparkeria* seems to have been more complete than in *Proterosuchus alexanderi*. This is indicated by the presence of a small projection of the laterosphenoid (figure 15*c*) absent in *Proterosuchus alexanderi*.

The trigeminal nerve (CN V) innervates a diverse group of tissues and muscles that include the regions of the nose, mouth, facial skin, cornea, teeth, palate and pharynx, among others, and is, therefore, the largest of the cranial nerves [64]. In *Euparkeria*, the margin of its foramen is formed by the prootic (figure 1*f*, CN V), except for the anterodorsal border, which was formed by the laterosphenoid (figure 15, CN V). The lateral surface of the prootic bordering the posterior region of the foramen is gently depressed, indicating the external position of the Gasserian ganglion in relation to the brain cavity (figure 2*e*, gr.ga). The ventral and anteroventral borders of the trigeminal foramen are formed by the ossification of part of the base of the embryonic pila antotica—the anterior inferior process of the prootic (figure 2*e*, aip).

As the suture between prootic and parabsisphenoid extends through the foramina for the abducens nerve (CN VI), the prootic and parabasisphenoid thus form the laterodorsal and ventromedial borders of these foramina, respectively (figure 2*a*, CN VI). The foramina for CN VI are located on the dorsum sellae, near its lateral margin at about its midheight. The lateral borders of the foramina are partially concealed in anterior view by the clinoid processes of the parabasisphenoid.

On the right-hand side of SAM-PK-7696, the foramen for the facial nerve (CN VII) is largely concealed by crest 1 of the prootic (figure 1*e*, cr1). On the left-hand side, crest 1 does not obscure the foramen (there appears to have been post-mortem distortion on the right-hand side), but the foramen sits in a groove formed by crest 1 anteriorly (figure 1*f*, CN VII; also seen on SAM-PK-5867, figure 9*c*). This groove extends dorsally and ventrally, marking the routes of the hyomandibular (figure 2*e*, CN VII_hym_) and palatine (figures 2*e* and 11*c*, CN VII_pal_) branches of CN VII, respectively. As described earlier, the hyomandibular branch of the facial nerve would have left the braincase from the posterodorsal part of the foramen, and continued posterodorsally towards the base of the paroccipital process, whereas the palatine branch would have exited the ventral part of the foramen, and extended ventrally and then medially along the posterior margin of the basipterygoid process [46,54–56].

As the medial wall of the otic capsule is mostly unossified, there are no foramina for the branches of the auditory nerve (CN VIII). The glossopharyngeal (CN IX), vagus (CN X), and likely also the accessory (CN XI) nerves exited the braincase through the metotic foramen (figures 1*e*,*f*, 3*a,f*, 6, 7*b*, 11*a,b* and 12*b*, mf). The presence of a shallow recess that closely resembles a foramen on the right-hand side of SAM-PK-7696 may indicate a separate and independent route for the CN XI (figure 5*a*, ?CN XI). The dorsal portion of the metotic foramen is somewhat narrower than its ventral portion, and the cranial nerves would have left the brain cavity through the former, while the perilymphatic sac would have been sited on the latter.

In squamates, the anterior branch of the hypoglossal nerve (CN XII) has smaller fibres and innervates the tongue, whereas the posterior branch is morphologically very similar to the first and second spinal nerves [55,66]. The hypoglossal nerve may leave the braincase as a single ramus, in which case the exoccipital is pierced by only one foramen. However, the presence of two independent foramina (figure 6*a*, CN XII_a_, CN XII_p_) indicates that the divergence between the branches took place before the CN XII exited the braincase in *Euparkeria*.

Foramina for the ethmoidal and ophthalmic arteries were found in *Proterosuchus alexanderi* [63], but no similar structures could be identified in *Euparkeria*, possibly due to the poorer preservation of the laterosphenoid. The vidian canals are preserved in all specimens of *Euparkeria* except SAM-PK-7696. The canals are not well preserved in any of the specimens although it is possible to verify that they make a simple connection with the hypophyseal fossa, perforating the basisphenoid ventrally (figures 11*a* and 12*f*, ica), posteromedial to the bases of the basipterygoid processes and extending anterodorsally into the fossa. The vena capitis dorsalis exited the brain cavity through a foramen located between the prootic, supraoccipital, parietal and laterosphenoid (figures 1*a,c*, 3*a*, 5*b*, vcd and 11*b*).

## 5. Discussion

In the light of the new data provided by CT scanning, and re-examination of all available material of *Euparkeria*, it is now possible to clarify a number of points of uncertainty remaining from previous studies regarding the braincase anatomy of *Euparkeria*. We thus address these points, roughly in the morphological order of description used above, in the discussion. Additionally, we examine and discuss *Euparkeria* in the broader context of diapsid braincase evolution, with our understanding again facilitated by the new data available to us. Furthermore, we suggest new directions for future studies of archosauriform and diapsid evolution.

### 5.1. Previous literature

#### 5.1.1. Basioccipital

The basioccipital of *Euparkeria* was described by Gower & Weber [42] as contributing to the basal tubera with ‘relatively long and slender’ [42, p. 373] projecting tongues of bone in comparison to ‘other earliest archosaurs’ [42, p. 373]. Examination of SAM-PK-5867 (figures 7*b* and 11*a*), however, shows that while the occiput of *Euparkeria* is overall more gracile than those of other crownward archosauriforms, the basal tubera nonetheless have similar proportions to those of, for instance, *Xilousuchus sapingensis* [51]. The basal tubera of *Xilousuchus* were described as similar to those of *Garjainia prima* and *Fugusuchus hejiapensis*, which, in turn, were considered ‘small, simple and ventrally projecting’ [51, p. 884] and ‘broad, flat and simple’ [51, p. 889] ventral projections respectively. Nesbitt [28] introduced a new character describing the nature of the anteroposterior thickness of the basioccipital portions of the basal tubera (character 106), in which non-pseudosuchian archosauriforms including *Euparkeria* show basal tubera which are ‘rounded and anteroposteriorly elongated’ [28, p. 89] (state 0, contrasting with the ‘bladelike and anteroposteriorly shortened’ basal tubera of some crocodile-line taxa). We agree with the scoring of Nesbitt [28] and find that the basal tubera of *Euparkeria* cannot be described as exceptionally long and slender among stem and early archosarus either in lateral or posterior view.

#### 5.1.2. Parabasisphenoid

Welman [47] labelled the cerebral branch of the internal carotid artery as extending into the parabasisphenoid through foramina located in the semilunar depression. As indicated in the previous section, the entrance foramina of the artery are located on the ventral surface of the bone, posteromedial to the base of the basipterygoid processes (figures 11*a* and 12*f*, ica). This is in agreement with the scoring of character 95 of *Euparkeria* in Nesbitt [28]. Welman [47] also stated that the basipterygoid processes of *Euparkeria* are more similar in shape to those of palaeognath birds than to those of dinosauromorphs and pseudosuchians. We found it difficult to assess this statement as little explanation of exactly what is regarded as similar is provided. However, the basipterygoid processes of *Euparkeria* have little particular resemblance with those of palaeognath birds. The processes of *Struthio camelus* (ZMB 2000 2769), with which Welman [47] made extensive comparisons, are short and laterally orientated (figure 19*a*, bt). They are highly pneumatic and anteroposteriorly elongated in cross section, with the long axis directed slightly ventrally. This general morphology is in fact quite similar to that of *Sphenosuchus acutus* [67], while the processes of *Coelophysis bauri* [28] are also small and anteroposteriorly elongate, but ventrally orientated and with their long axes dorsally directed.

**Figure 17.**
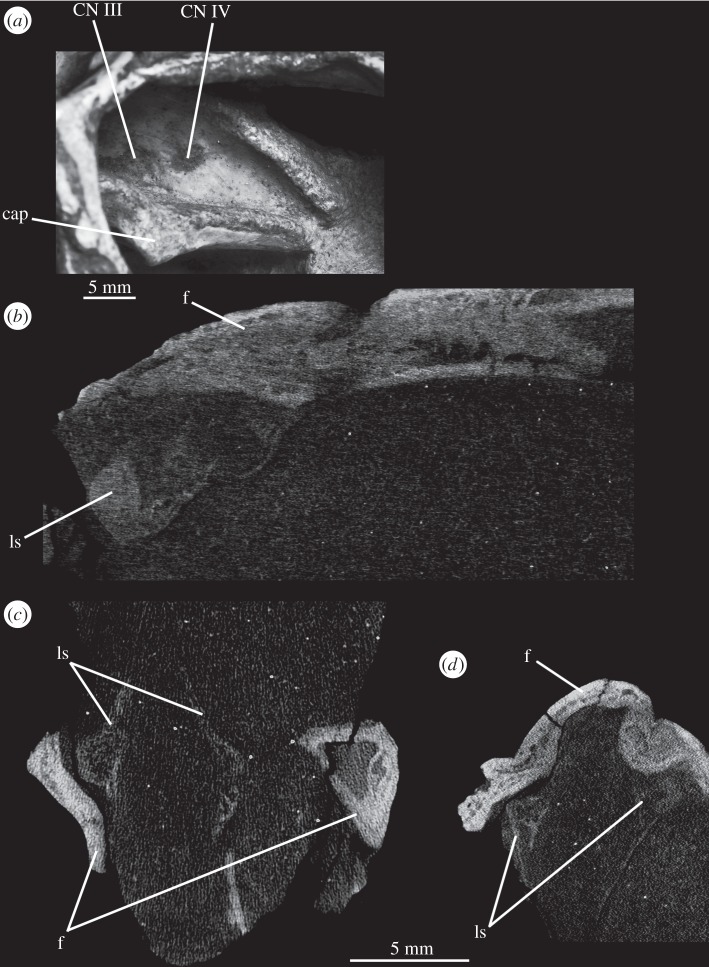
Laterosphenoid of SAM-PK-5867 (*a*) in left dorsolateral view, and CT slices on laterosphenoids of UMZC T.692 in (*b*) right lateral, (*c*) dorsal and (*d*) posterior view. For abbreviations, see table 1.

The route of the suture line between the prootic and parabasisphenoid described earlier contrasts with that described by Cruickshank [45], who indicated that the parabasisphenoid formed part of the posterior margin of the fenestra ovalis (a ‘small posteriormost process in front of the ventral ramus of the opisthotic’ [45, p. 684]) and a large part of its anterior margin. The suture between the prootic and the parabasisphenoid was identified in the CT scans as extending directly posterodorsally to anteroventrally from the anteroventral margin of the fenestra ovalis (figures 3*e* and 6*c*). However, the basisphenoid does have a small dorsal process laterally that forms the anteroventral border of the fenestra ovalis (figures 1*e*, 5*a*). The suture identified by Cruickshank [45] is in fact the groove for the palatal branch of CN VII, and the ‘posteriormost process' indicated by Cruickshank is absent. Ewer [14] indicated the parabasisphenoid–prootic suture line broadly correctly, with the parabasisphenoid ascending dorsally as a short posterior process anterior to the fenestra ovalis, then descending again along the groove for CN VII. Gower & Weber [42] excluded the dorsal process of the parabasisphenoid that contributes to the fenestra ovalis from that element entirely, indicating the prootic–parabasisphenoid suture line as extending anteroposteriorly; the posterior portion of the line indicated by Gower & Weber [42] is likely a crack, with the fragment of bone immediately dorsal to this crack also being part of the parabasisphenoid; it is also possible to identify the ascending process of the parabasisphenoid on the left side of SAM-PK-5867. A tall posterior ascending process of the parabasisphenoid was depicted by Dzik [68] in the braincase of *Silesaurus*. Upon examination of the relevant specimens, however, it is difficult to verify exactly the contribution of the parabasisphenoid to the fenestra ovalis. On the right-hand side of ZPAL Ab III/364 the process seems robust and tall, but on the left side it is was not found. There are no signs of processes on a second *Silesaurus* specimen, ZPAL Ab III/361. *Stagonolepis olenkae* (ZPAL AbIII/466/17, right-hand side) also shows the parabasisphenoid participating in the anteroventral border of the fenestra ovalis, but the prootic–parabasisphenoid suture is difficult to follow anterior to this and an ascending process may have been absent.

We confirm the statement of Gower & Weber [42] that the lateral depression is formed entirely by the parabasisphenoid (figure 3*e*) and there is no participation of another element, such as the alaparasphenoid [47] or the prootic [46]. We also confirm that the lateral depression is not connected to any cavity within the parabasisphenoid itself or within the basioccipital. This is confirmed not only upon examination of SAM-PK-7696, but also of SAM-PK-5867 and UMZC T.692, and is thus not a result, as suggested by Gower & Weber [42], of individual variation. However, the braincase as a whole is lightly constructed; while this is exaggerated to some extent in SAM-PK-7696 due to acid preparation, the entire braincase is formed of trabeculate bone. In UMZC T.692 and SAM-PK-6047A, the preserved parts of the basipterygoid processes appear hollow in the CT scans; this may be either because the delicate trabeculae were damaged or because the scan made was not sensitive enough to detect them. We were not able to locate any foramen or channel connecting these or other internal spaces to the outside (discussed later). With the reevaluation of the dorsal extent of the parabasisphenoid and its subsequent contribution to the anterior part of the lateral braincase wall, our perception of the roles of the parabsisphenoid and prootic is also altered and is discussed in a later section. Other aspects of the lateral depression and of potentially pneumatic features, such as its homologous nature with the anterior tympanic recess (ATR), will also be discussed in a later section.

Scans of SAM-PK-5867 and UMZC T.692 confirm the conclusions of Gower & Weber [42] that *Euparkeria* possesses no intertuberal plate, in contrast with Nesbitt [28], who scores the intertuberal plate as present and arched anteriorly. The anterior border of the medial pharyngeal recess of *Euparkeria* is, however, arched, and we thus suppose Nesbitt [28] must have mistaken this border for an intertuberal plate; the intertuberal plate of some taxa, e.g. *Arizonasaurus babbitti* (see [69]), is indeed both present and arched anteriorly, but the distribution of this morphology is much more restricted than indicated by Nesbitt [28].

#### 5.1.3. Exoccipital

Gower & Weber [42] tentatively described the exoccipitals as not contacting medially. This was reconsidered in Gower [57], and based on the scoring of this character for *Euparkeria*, Nesbitt [28, p. 91] stated that ‘[p]lesiomorphically among archosauriforms, the exoccipitals meet along the midline preventing the basioccipital from participating in the endocranial cavity’, also scoring *Euparkeria* as having contacting exoccipitals. By tracing the suture lines in CT data, we can confirm that the exoccipitals did not meet at the midline in SAM-PK-7696, with the ventral border of the foramen magnum thus formed by the basioccipital (figure 6*a*). In SAM-PK-5867, the exoccipitals also do not meet, but they approach each other more than in SAM-PK-7696 (figure 7*b*), and we consider this to be the result of the lateral compression suffered by the specimen. In UMZC T.692, however, the right exoccipital extends further medially than in SAM-PK-5867 and almost reaches the midline (figures 11*b*, 14*b*), but does not contact its antimere. We can also confirm that there are two foramina for CN XII in both SAM-PK-7696 and SAM-PK-5867, with a third foramen in the region of SAM-PK-7696 potentially attributable to CN XI (figure 5*a*). We agree with Gower & Weber [42] that CN X would have left the braincase via the metotic foramen as in all modern amniotes without a proper vagus foramen, *contra* Ewer [14], who identified the upper portion of the CN XII foramina as being for CN X.

#### 5.1.4. Opisthotic

The suture between opisthotic and exoccipital was identified with the assistance of CT scans (figure 6*a*), and it can be confirmed that the exoccipital is restricted to the pillar between the metotic foramen and the foramen magnum, not participating in the paroccipital process. We can confirm that Cruickshank [45] and Welman [47] were correct in their identification of a delicate bony bridge connecting the distal end of the ventral ramus of the opisthotic and the parabasisphenoid on the right-hand side of SAM-PK-7696 (figures 2*e* and 3*d,f*, bb). We also identify this feature on the right-hand side in SAM-PK-5867 (figure 9*b*, bb). Damage prevents its identification on the left-hand side in both specimens and in UMZC T.692 (as discussed later).

#### 5.1.5. Prootic

Gower & Sennikov [51] reported a small ridge on the anterior inferior process of the prootic of *Euparkeria*, but further examination of UMZC T.692 led Gower & Weber [42] to reidentify it as a preservational artefact and to consider the ridge absent in *Euparkeria*. The absence of such a ridge was also identified by Nesbitt [28] as a synapormorphy of Archosauria (character 94). We confirm, however, that the ridge is indeed present in *Euparkeria* in SAM-PK-7696 (figure 2*f*, rd) and on the left-hand side of SAM-PK-5867 (although poorly preserved; damage prevents accurate assessment in UMZC T.692 and in SAM-PK-6047A the prootic is not preserved) and marks the ventral edge of the depression for the Gasserian ganglion. Such a ridge also forms part of the raised border of the depression for the Gasserian ganglion in other archosauriforms (e.g. *Trilophosaurus buettneri* [70]) and in most achosaur taxa where the entire border of the trigeminal foramen is well known, e.g. *Dysalotosaurus* [56], *Stagonolepis olenkae* (ZPAL AbIII/466/17) and *Adeopapposaurus mognai* (PVSJ 568). We also disagree that this feature is absent in *Erythrosuchus* (BP/1/3893; *contra* [28,62]), although the ridge is much less prominent than in crown taxa. The phylogenetic informativeness of this feature is thus, in our opinion, doubtful.

Two separate foramina for the palatine and hyomandibular branches of the facial nerve were identified first by Ewer [14], and this was followed by Cruickshank [45]. We agree with Gower & Weber [42] that only one foramen is present in UMZC T.692, and could also identify only one opening for CN VII in SAM-PK-7696 (figures 1*e*,*f* and 3*b*,*e*) and on the left-hand side of SAM-PK-5867 (the right-hand side is too damaged for assessment). As correctly noted by Gower & Weber [42], the area identified as housing the palatine foramen by Ewer [14] and Cruickshank [45] is simply a blind depressed area on the lateral surface of the parabasisphenoid. Separate foramina for the hyomandibular and palatine branches of the facial nerve are not found with certainty in any taxa outside Theropoda, and it appears safe to state that such a separation did not occur until much later in archosaurian evolutionary history, and that there is no individual variation in *Euparkeria* (*contra* the suggestion of Gower & Weber [42]).

#### 5.1.6. Supraoccipital

Gower & Weber [42, p. 379] stated that ‘[i]f the medial suture between the prootic and supraoccipital has been correctly identified, then the posterodorsal end of the floccular recess just extends onto the supraoccipital on the left of UMZC T.692, and the broken surface exposed above the recess on the right side represents the prootic surface for articulation with the supraoccipital’. We disagree that the floccular fossa extends onto the supraoccipital, but we agree that the suture between prootic and supraoccipital should extend just dorsal to the recess. We also find it difficult to understand how, if the floccular fossa extends dorsally onto the supraoccipital, the articular surface of the prootic would be exposed dorsal to the recess. Gower & Weber [42] also state that ‘[a] shallow groove on the left side immediately anterior to the floccular recess is interpreted as indicating the probable path of the middle cerebral vein’. What we identify as the hollow for the transverse sinus, more than the middle cerebral vein itself (as mentioned earlier), is not preserved in UMZC T.692. The area indicated by Gower & Weber [42] instead corresponds to the anterior part of the subarcuate fossa which has been anteroposteriorly compressed, creating the appearance of a shallow groove.

Welman [47] identified an epiotic bone anterior to the dorsal part of the base of the paroccipital processes in both in SAM-PK-5867 and SAM-PK-7696, though only indicated the suture between it and the opisthotic and prootic in the latter. We can find no evidence for a separate ossification in this region both using CT data and on re-examination of the specimens. The anterolateral margins of the supraoccipital in SAM-PK-5867 appear to be more rounded and extended than in SAM-PK-7696, and this could be potentially indicative of an ossification separate from the supraoccipital in this position that is absent in SAM-PK-7696. However, we can find no sutural distinction between these areas of the skull roof and the rest of the supraoccipital in SAM-PK-5867, and these differences in shape may be more readily explained by mediolateral compression and the articulation with the interparietal and parietal in SAM-PK-5867. We thus find no good evidence for the existence of a separate epiotic in *Euparkeria*.

#### 5.1.7. Fenestra ovalis and metotic foramen

Welman [47] used the size of the fenestra ovalis as a synapormorphy uniting *Euparkeria* and birds with the exclusion of dinosaurs. We agree with Gower & Weber [42] that, without quantification of ‘large’ or ‘small’, it is difficult to compare the state in *Euparkeria* with that in other taxa. Taxa on both the archosaur stem, e.g. *Mesosuchus browni* (SAM-PK-6536), and on the crocodile line, e.g. *Stagonolepis olenkae* (ZPAL AbIII/466/17), show a fenestra ovalis just as extensive as that of *Euparkeria*, and we find Welman's [47] conclusions thus doubtful. We also agree with Gower & Weber [42] that there is no fenestra pseudorotunda, rather only an unsubdivided metotic foramen. There is thus no metotic strut—the structure which would subdivide the foramen [42,52], formed from the metotic cartilage and separating CN IX from CN X [71]. Although neither feature is present in *Euparkeria*, we also point out that the metotic strut is a distinct feature from the lateral ridge of the exoccipital (which separates CN XII_a_ from CN XII_p_), *contra* Nesbitt [28].

Character 114 of Nesbitt [28] synonymizes the lateral ridge of the exoccipital of Gower [57] with the metotic strut of theropods. However, these two structures differ in their topological position, and cannot be homologous. The term ‘metotic strut’ appears to have been introduced into the fossil avialan literature by Witmer [72] to refer to the ossification of the metotic cartilage, a structure related to the formation of the recessus scalae tympani and located between the nerves glossopharyngeal (CN IX) and vagus (CN X) [71]. The lateral ridge of the exoccipital as used by Gower [57], is, by contrast, located between the anterior and posterior branches of the hypoglossal (CN XII) nerve. Gower [57] was clearly aware of the developmental context in which the term ‘metotic strut’ was coined and used, for it had been part of the base of the argument of Gower & Weber [42, section 3a-V] for the absence of a fenestra pseudorotunda in *Euparkeria*. We thus disagree with the synonymization proposed by Nesbitt.

#### 5.1.8. Inner ear

We are also able to shed light on several aspects of the anatomy of the inner ear. Gower & Weber [42, p. 389] state that ‘there is no clearly ossified differentiation between the canalicular and cochlear parts of the inner ear’ in UMZC T.692, but we do find a lagenar crest to be present in SAM-PK-7696 (figure 5*a,b*). However, we agree with Gower & Weber [42] that evidence regarding shape of the cochlea is inconclusive. The otic capsule of diapsids is not extensively ossified as in mammals [73], and thus the exact shape and length of the cochlea cannot be assessed based on osteology alone, and *Euparkeria* is no exception. We also agree with Gower & Weber [42, p. 389] that ‘part of the anteroventral limit of the vestibule can be detected in *Euparkeria* as a subhorizontal ridge in UMZC T.692, on the medial surface of the braincase immediately above the facial foramen’. The lagenar recess is formed equally by the basioccipital and the basisphenoid medially, and thus differs from the description of Welman [47]. Gower & Weber [42] were unsure if the unossified gap would indicate the ventralmost part of the lagenar recess, but, as we can confirm that it does (discussed later), the orientation of the cochlea can be more precisely reconstructed based on the lagenar crests and the unossified gap as being straight ventral. Nesbitt [28] (character 118) identified *Euparkeria* as having no well-defined lagenar recess. This is, however, based on Gower [57], which is in turn based on the reluctance of Gower & Weber [42] to identify the unossified gap as part of the lagenar recess.

A marked notch on the right medial wall of the opisthotic of SAM-PK-7696 is likely an artefact. The surfaces of the ventral ramus of the opisthotic around this notch are damaged on both sides, and CT scans show that disarticulated fragments of bone are attached to them with what appears to be glue (figure 16*e,f*). However, the ventralmost portion of the medial surface of the ramus does appear to bear a gently rounded broad notch, and a similar structure is seen on the left-hand side of SAM-PK-5867. This leads to the conclusion that the lateral border of the perilymphatic foramen is identifiable, but it is more ventrally located than the original area indicated by Gower & Weber [42]. The perilymphatic foramen can be confirmed to lack a bony medial border, as is also the case in *Sphenodon* (ROM R9298, figure 20). However, the structures of *Euparkeria* and *Sphenodon* differ in several respects. Firstly, the perilymphatic foramen in *Euparkeria* would have been more laterally located than in *Sphenodon*. Secondly, the dorsal and ventral borders of the foramen in *Euparkeria* are less extensively ossified, and altogether the medial extension of the ossified part of the ramus in *Sphenodon* is greater. Furthermore, the axis of the ventral ramus is slightly twisted in *Sphenodon*, so that the perilymphatic duct would have extended in an anterolateral to posteromedial direction, whereas in *Euparkeria* it is straighter, and the duct would thus have extended roughly anteroposteriorly. Finally, in *Sphenodon*, the ventral half of the perilymphatic foramen is formed by the basioccipital, while in *Euparkeria* it is formed by the opisthotic.

#### 5.1.9. Unossified gap

The open area between the ventralmost tip of the ventral ramus of the opisthotic, the anterodorsal part of the basioccipital, and the posterodorsal region of the parabasisphenoid was identified by Cruickshank [45] as the lagenar recess and by Welman [47] as the fenestra pseudorotunda. Gower & Weber [42] termed this structure as an ‘unossified gap’ and homologized it with similarly positioned unossified gaps of *Sphenodon* and other diapsids. Gower & Weber [42] corrected Welman's [47] interpretation of the presence of a fenestra pseudorotunda, as this structure relates to the bony subdivision of the metotic foramen and the formation of a true recessus scalae tympani. The metotic foramen of *Euparkeria* is not subdivided, and thus no fenestra pseudorotunda is present.

Although Gower & Weber [42] indicated that this unossified gap could represent part of the lagenar recess, there remained some uncertainty. The specimen UMCZ T.692 provides only a medial and a damaged lateral view of the braincase, and Gower & Weber [42] had only a cast of SAM-PK-7696 and the work of Cruickshank [45] at their disposal. With CT scans of SAM-PK-7696 and of SAM-PK-5867 at hand, in the light of the growing literature on braincase and neuroanatomy of fossil archosaurs [56,60,74] and based on comparisons to extant lepidosaurs [55], we confirm that this space represents the ventralmost part of the lagenar recess.

We are also able to confirm that the bony bridge separating the aperture of the gap from the margin of the fenestra ovalis is mostly formed by a thin, but marked posterolateral process of the basisphenoid (figure 3*d,f*). What unfortunately remains unclear is whether there is a minor anterior contribution of the opisthotic. On the right side of SAM-PK-7696, the cortex of the anterior surface of the distal part of the ventral ramus of the opisthotic seems to have been worn away during preparation (figure 1*f*), together with the medial border of the unossified gap formed by basioccipital and basisphenoid, whereas on the left side this structure is not preserved. The left side of SAM-PK-5867 is severely damaged and on the right side only the posterolateral process of the basisphenoid remains. If the opisthotic contributed to this bridge, then it was probably just a small eminence for the articulation with the basisphenoid.

#### 5.1.10. Semilunar depression

On the right-hand side of SAM-PK-7696, immediately anterior to the unossified gap there is a clear depression on the lateral surface of the parabasisphenoid contribution to the basal tuber (figure 1*e*). The posterodorsal border of this depression is open. The anteroventral border is delimited by a crest of bone of the parabasisphenoid on the proximal end of the basal tuber. This structure was identified as the semilunar depression by Gower & Weber [42]. The term was introduced by Evans [46, p. 186] for a similar feature in *Prolacerta* and *Mesosuchus* and subsequently identified in other archosauriforms [51,61,62]. Evans [46, fig. 7] illustrated the above-mentioned feature in SAM-PK-7696, but did not label it. We are not able to locate this structure with certainty in SAM-PK-5867 because the basal tubera of the parabasisphenoid are damaged. The form of the semilunar depression in SAM-PK-7696 differs notably from other taxa, being much more pronounced and more dorsally, as opposed to laterally, open than in *Prolacerta* (BP/1/2675), *Proterosuchus alexanderi* (NMQR 880), *Osmolskina* (ZPAL RV/413 and ZPAL RV/424) and *Dorosuchus* (PIN 1579/62), but this may be exaggerated by loss of the posteroventralmost part in this specimen.

Of the putative functions suggested for this structure, the function as an articular facet for the ventral ramus of the opisthotic can probably be excluded as in articulated specimens of both *Euparkeria* (SAM-PK-7696) and *Proterosuchus goweri* (NMQR 880) the semilunar depression is exposed. Furthermore, in *Prolacerta* (BP/1/2675) the ventral ramus is somewhat bent at its mid-portion, so that the distalmost part is clearly ventrally directed and could not have articulated with the semilunar depresion. Likewise, Evans [46, fig. 7] illustrated the ventral ramus of *Euparkeria* as anteroventrally directed towards the semilunar depression, but this is inaccurate. The ventral ramus of the opisthotic is only slightly anteriorly directed and bends very gently, extending ventrally until its distal tip (figure 5*a*); no articulation with the semilunar depression is present. The alternative functional suggestion of Evans [46] for the semilunar depression is to serve as a ‘line of attachment for connective tissue filling in the lower part of the overlying fenestra ovalis'. Considering this and the definition of Gower & Weber [42] that a ‘lateral opening between opisthotic, parabasisphenoid and basioccipital […] represents an unossified area […] that would probably have been covered by cartilage in life’, then it would make the semilunar depression of Evans [46] the anteroventral part of the unossified gap of Gower & Weber [42]—or the parabasisphenoid contribution to the unossified gap. However, a similar depression appears to be absent in crown archosaurs, non-archosauriform archosauromorphs and *Sphenodon* (ROM R9298, figure 20*c*,*d*), although an unossified gap is often present. With the absence of a homologue in extant taxa we cannot be certain as to the function of the semilunar depression.

#### 5.1.11. Pneumatization

No true pneumatic cavity was found in the sense of an internal space within a bone connected through a foramen to other external spaces such as the middle ear cavity or the pharyngeal sinus. However, CT scanning revealed trabeculate, rather than compact, bone histology in the braincases of *Euparkeria*. The pneumatic system often includes shallow recesses that do not necessarily perforate adjacent bones (hereafter termed pneumatic sinuses), and in the case of *Euparkeria*, these may include the ventral median pharyngeal recess (figures 1*d*, 7*b*, 11*a*, 12*f* and 14*b*,*d*, mpr) and the lateral depression of the parabasisphenoid (figures 2*e*,*f*, 3*e*, 5*a,b*, 9*a* and 14*e*, ld). The median pharyngeal recess (basisphenoid recess of Witmer & Ridgely [75]) is present in a number of non-archosauriform reptiles such as *Captorhinus laticeps* [54], *Youngina* [50] and *Prolacerta* [46], whereas the lateral depression is probably homologous with the ATR of theropods and birds (as discussed later; *sensu* Witmer & Ridgley [75]), but unlike in coelurosaurs [75], it shows no pneumatization of the surrounding bones. Nesbitt [28] scored a median pharyngeal recess as absent in *Euparkeria* (character 107), but his definition of this structure seems to correspond to a pronounced depression at the anterior extreme of the ventral fossa at the midline. Such a depression is indeed absent in *Euparkeria*, although appears to also be absent in some of the taxa where this is scored as present by Nesbitt [28] (e.g. *Turfanosuchus dabanensis* [76]). We would advocate using a different nomenclature (e.g. pronounced midline fossa at anterior of median pharyngeal recess) to describe this more pronounced recess to avoid confusion with the more broadly applicable ‘median pharyngeal recess'.

Gower & Weber [42] described the deep lateral depression of the parabasisphenoid as not being homologous with the ATR of birds (*contra* [47]), and Nesbitt [28] restricted the presence of a true ATR to dinosauromorphs (character 101). However, while no pneumatic sinuses leading from the lateral depression can be identified, we find that the lateral depression in *Euparkeria* corresponds topologically to the ATR of dinosaurs and birds. In dinosaurs and birds, the ATR arises in the region of the internal carotid foramen, between the alaparasphenoid and the basisphenoid. The facial nerve exits the braincase within or just posterior to the ATR and its palatine branch traverses the recess [72,75]. Similarly, the ATR of *Euparkeria* is located on the lateral surface of the parabasisphenoid, posterodorsal to the basipterygoid process and just ventral to the exit of the CN VII, and the palatine branch of the nerve also crosses the area. In *Euparkeria*, the internal carotid artery does not enter the parabasisphenoid in the ATR area, but in taxa where the internal carotid artery pierces the bone laterally instead of ventrally it does so in this same region (e.g. [77], as discussed later). The ATR identified in *Silesaurus* lacks pneumatic sinuses [28,68], but is still classed as an ATR [68] and is extremely similar both in terms of morphology and topology to the lateral depression of *Euparkeria*. It is also located on the lateral surface of the parabasisphenoid, posterodorsal to the basipterygoid process and ventral to the foramen of the CN VII and on the course of its palatine branch. It is, however, worth noting that the ATR of *Silesaurus opolensis* is deeper and larger than that of *Euparkeria*, with a lateral expansion of the parabasisphenoid marking its anterior limit [77].

We feel that the only meaningful distinction to be made between the ATR and the lateral depression is whether pneumatic sinuses are present leading off into the braincase wall from the depression. Following this distinction, both *Silesaurus* and *Euparkeria* would have a lateral depression while some dinosaurs would show an ATR. A more straightforward way of describing this difference, reflecting better the homology of these structures, may be an ATR lacking pneumatic sinuses versus one showing pneumatic sinuses. While we understand that caution is warranted in homologizing pneumatic structures [72], we do not see justification for homologizing the recess of *Silesaurus* (and other taxa lacking pneumatic pneumatic sinuses, e.g. *Lewisuchus* [78]) with that of dinosaurs if that of *Euparkeria* is not homologized similarly. We would advocate two separate homology statements: one homologizing the recess of *Euparkeria*, dinosaurs and *Silesaurus* based on its topological correspondence, and a second homologizing the presence of pneumatic pneumatic sinuses (i.e. the condition seen in theropods). We also note that a very similar structure, which we would homologize with the lateral depression of *Euparkeria*, is present in some non-crocodylomorph pseudosuchians (e.g. *Stagonolepis olenkae* [77], *Prestosuchus chiniquensis* [79], *Shuvosaurus inexpectatus* [80,81], *Postosuchus* [57]), and thus the presence of a lateral depression (=ATR) does not necessarily support avemetatarsalian affinities for *Euparkeria*.

#### 5.1.12. Laterosphenoid

The presence or absence of an ossified element forming the anterior braincase wall of stem archosauriforms was a topic of some uncertainty until the presence of such ossifications was demonstrated in *Euparkeria* and *Proterosuchus alexanderi* by Clark *et al*. [63]. However, at the time of that publication, further preparation to reveal the laterosphenoids of the holotype of *Euparkeria* was not complete, and an apparent disarticulated laterosphenoid in SAM-PK-7696 was relied upon to describe the element. On inspection of SAM-PK-7696, we find it difficult both to confirm the identification of the element as a laterosphenoid and to identify morphological features thereof with certainty. Examination of the laterosphenoids of SAM-PK-5867 and UMZC T.692 provides confirmation of the morphology of this element in specimens where there is no doubt regarding the identification as a laterosphenoid.

**Figure 18.**
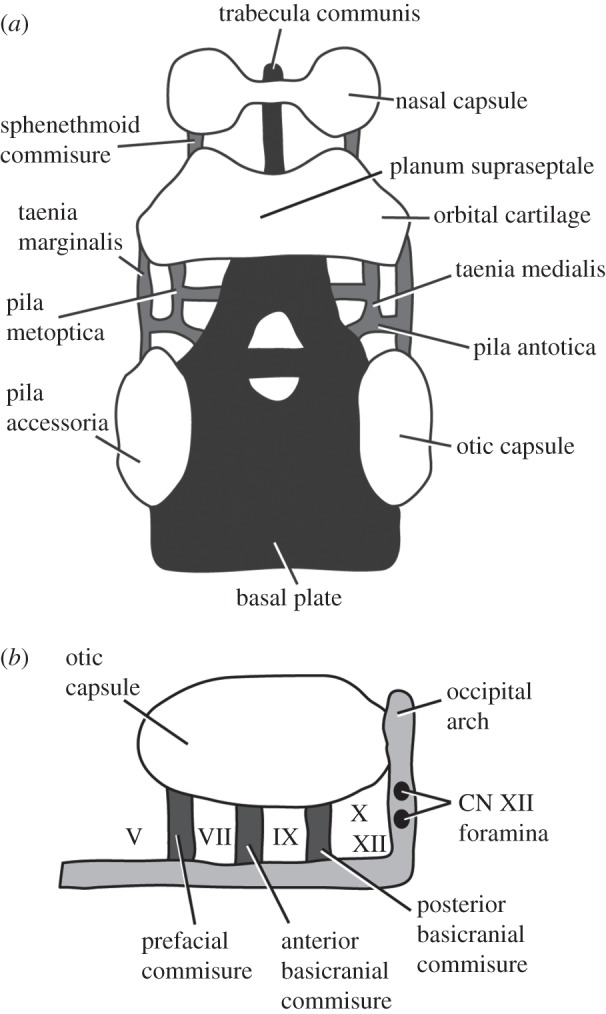
Schematic drawings of the lizard chondrocranium: (*a*) dorsal view, late stage, illustrating connections between sensory capsules and basal plate; (*b*) left lateral view, showing otic capsule and connections to basal plate. Both redrawn from [84, fig. 1.2]. For abbreviations, see table 1.

The first usage of the term ‘laterosphenoid’ was to refer to the anteriormost ossification of the braincase of crocodilians [63], but subsequent usage of the term included a non-homologous ossification in snakes, a partially homologous ossification in non-ophidian lepidosaurs, and a probably homologous structure in birds. In fact, the developmental definition of a laterosphenoid was based on the embryology of non-ophidian lepidosaurs, a group in which this element seldomly ossifies [82]. According to this, the laterosphenoid represents the ossification of part of the embryonic pila antotica (figure 18), a cartilaginous structure located between the exits of CN III and CN IV anteriorly and of the CN V posteriorly [82–84]. In addition to the laterosphenoid, another element may ossify (or just calcify) in the anterior braincase wall of non-ophidian lepidosaurs: the orbitosphenoid. The orbitosphenoid is formed by the ossification of the pila metoptica (which forms the anteriormost part of the embryonic braincase, between CN II anteriorly and CN III and IV posteriorly) with some contribution from the taenia medialis—which connects the pila metoptica to the structure supporting the olfactory bulb, the planum supraseptale [82] (figure 18). By contrast, the laterosphenoid of crocodilians does not quite conform to this definition. It is composed of the pila antotica, the pila metoptica and part of the taenia medialis [82,85]. If preference is given to the first usage, the laterosphenoid and orbitosphenoid of non-ophidian squamates can thus be looked upon as ossifications of subsets of the ‘true’ laterosphenoid.

The ossifications of *Euparkeria* and *Proterosuchus alexanderi* appear to fundamentally conform to the crocodilian condition, and thus presumably to include all three embryonic elements. However, the laterosphenoids of *Euparkeria* and *Proterosuchus alexanderi* differ from those of crocodilians in that a slender process is present, markedly separating the foramen of CN III and CN IV from that of CN II (figure 15, sp). By contrast, in crocodilians, the separation of these nerves by the laterosphenoid is made by a very modest process, the ventral portion of which is completed by a dorsal extension of the cultriform process of the parabasisphenoid (figure 21*a–c*). These differences can be interpreted as indicating a greater degree of ossification of the pila metoptica in *Euparkeria* and *Proterosuchus alexanderi* than in crocodilians. Furthermore, the foramina for the olfactory and optic nerves are only separated by a brief contact of the right and left laterosphenoids in crocodilians (figure 21*d*).

**Figure 19.**
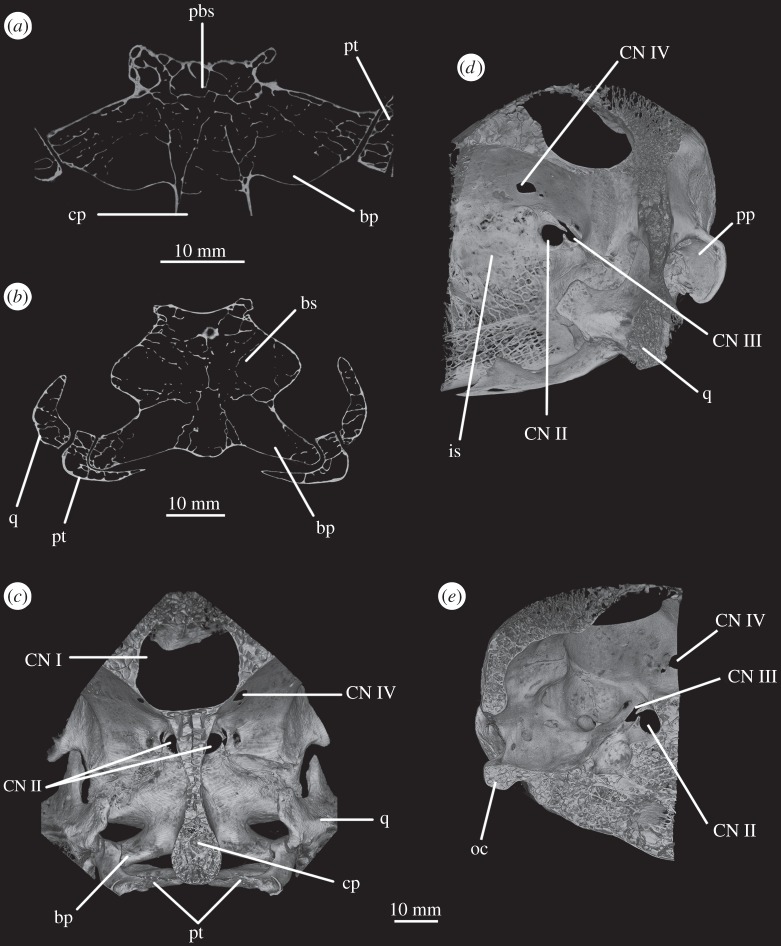
Braincase of *Struthio camelus* (ZMB 2000 2769): (*a*) transverse and (*b*) coronal CT cut of the anterior part of the braincase showing morphology of basipterygoid processes; CT reconstructions of braincase in (*c*) anterior (with posterior part of braincase removed for clarity), (*d*) left anterolateral and (*e*) left medial views. For abbreviations, see table 1.

By contrast, in *Euparkeria*, and yet further so in *Proterosuchus alexanderi*, the contact is more extensive and the laterosphenoids of the fossil taxa extend further anteriorly between the orbits than the ossification in crocodilians, reaching as far as the anterior third of the orbit (in crocodilians the laterosphenoid does not reach the middle of the orbit, e.g. *Alligator* sp. specimen 238 of the Biological Sciences Collection of the University of Birmingham, UK). Such an anterior extension suggests a greater degree of ossification of the taenia medialis than in crocodilians, and also potentially that another embryonic element may, at least in part, be involved in the formation of the laterosphenoid of *Euparkeria* and *Proterosuchus alexanderi*, namely the planum supraseptale (figure 18). The planum supraseptale results from the fusion of the embryonic orbital cartilages and, as mentioned earlier, supports the forebrain. The planum supraseptale may form a ventral keel, the interorbital septum, to connect to the basal plate and together interorbital septum and planum supraseptale are considered to ossify as a third element, the sphenethmoid [82,84].

Sphenethmoids are common ossifications identified in reptiliomorphs close to amniotes such as diadectomorphs [86,87], and also in basal reptilians such as captorhinids and parareptiles [54,88,89] that are thought to be subsequently lost in saurians [90]. Although in many instances the sphenoid ossification does not ossify farther posteriorly at the anterior region of the braincase (e.g. [91]), the term ‘sphenethmoid’ does include ossifications that would in theory comprise parts of the laterosphenoid—the pila antotica, the pila metoptica and the taenia medialis [90,92]. Thus, the sphenethmoid would be homologous to the laterosphenoid of stem archosaurs and crocodilians. As revealed by the CT scans of SAM-PK-5867 and UMCZ T.692, ossifications of the anterior braincase wall are very thin and delicate structures that can be easily prepared away and it may well be that such structures are indeed present in other basal diapsids. In fact, a sphenethmoid has been identified in the diapsid of uncertain affinities *Elachistosuchus huenei* [93] and also tentatively in *Youngina* [94] (although it was not mentioned by Gardner *et al*. [50]).

The anterior braincase wall ossification of some basal pseudosuchians (e.g. *Stagonolepis olenkae* [77], *Shuvosaurus* [80,81]) appears to have been more similar to that of *Euparkeria* and *Proterosuchus alexanderi* than to that of modern crocodilians, in lacking a contact between the cultriform process and the slender process. However, other basal pseudosuchians do show such a contact (*Stagonolepis robertsoni* [72], *Desmatosuchus spurensis* [95]), though whether the parabasisphenoid formed part of the margin of the CN II foramen is not clear. The anterior braincase wall of other fossil taxa closer to (e.g. *Prestosuchus* [79]) or within (e.g. *Sphenosuchus* [67]) Crocodylomorpha are more similar to that of crocodilians in that the ventral border of the foramen of CN II is formed by the basisphenoid.

The condition in extant birds is more similar to the one found in *Euparkeria* and *Proterosuchus alexanderi* than to that in crocodilians, in that all the embryonic structures (namely the pila antotica, pila metoptica, taenia medialis, planum supraseptale and interorbital septum) ossify [82,84] (figures 19 and 22). However, in avian terminology, instead of sphenethmoid it is more common to find the terms ‘interorbital septum’ and/or ‘mesethmoid’ for the ossifications anterior to the foramen of CN II [96,97]. The presence of these ossifications in dinosaurs is well documented in derived ornithischians [65], sauropods [98–100] and theropods [60]. An orbitosphenoid distinguished from the laterosphenoid is often described, although in most cases sutures are difficult to identify.

**Figure 20.**
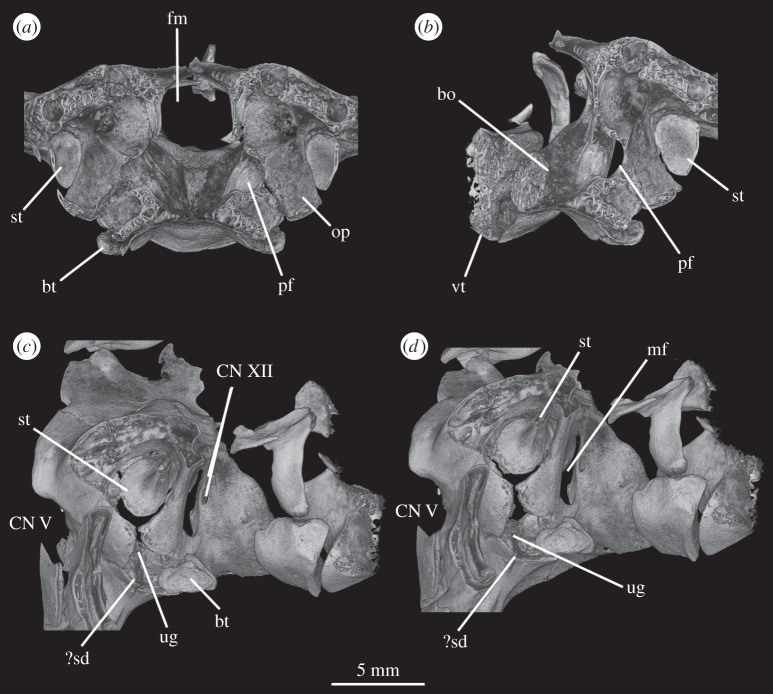
CT reconstructions of braincase of *Sphenodon punctatus* (ROM R9298): (*a*) anterior view in transverse cross section, (*b*) anteromedial view in cross section, (*c*) left lateral view and (*d*) ventroposterolateral view. For abbreviations, see table 1.

**Figure 21.**
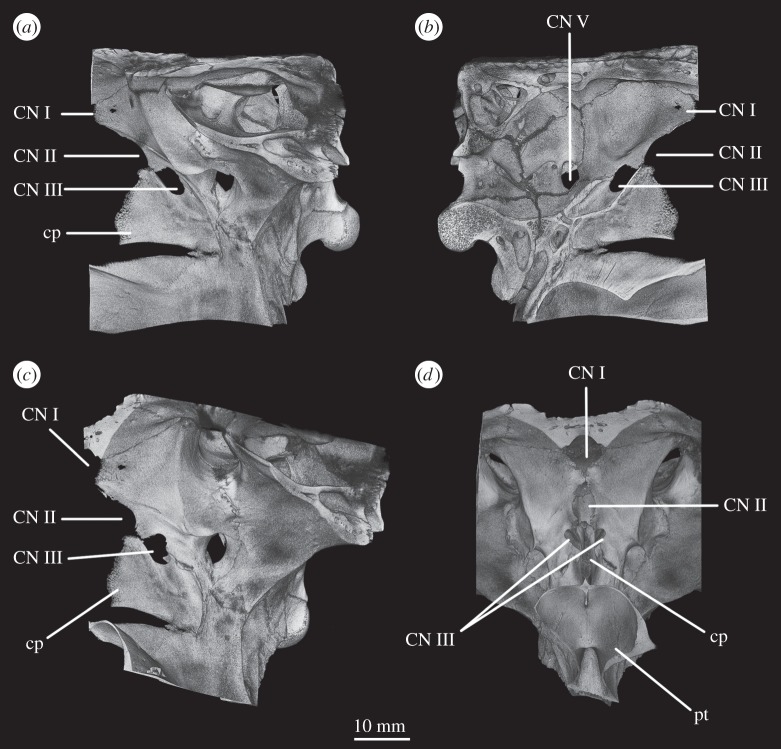
CT reconstructions of braincase of *Osteolaemus tetraspis* (ZMB 23467) in (*a*) left lateral, (*b*) left medial, (*c*) left anterolateral, and (*d*) anterior views. For abbreviations, see table 1.

A laterosphenoid is documented for a number of less derived crown taxa [28], but well preserved and complete elements suitable for a more detailed analysis are still somewhat rare. The laterosphenoid of *Heterodontosaurus tucki* appears similar to that of some pseudosuchians [101], in that a short spur makes the contact with the basisphenoid. However, the contribution of the basisphenoid to the foramen of CN II is unclear. The degree of ossification, as well as the anterior extension of this structure, seems to include not only the pila antotica, but also the pila metoptica and taenia medialis—but not the planum supraseptale. A distinction is made between laterosphenoid and orbitosphenoid in figs 2B and 15B of Norman *et al*. [101], but no description is provided.

On the other hand, the laterosphenoids of *Coelophysis bauri* [28], *Lesothosaurus diagnosticus* [20], *Tawa hallae* [26], as well as those of basal ornithopods *Dysalotosaurus* [56], *Thescelosaurus neglectus* [102] and *Hypsilophodon foxii* [103] are strikingly different. In these taxa, the ossification seems to be restricted to the posterior part of the pila antotica only. As a consequence, the exits of the cranial nerves I–IV are not represented by foramina and there is also no contact to the cultriform process of the parabasisphenoid. A foramen for CN III is identified for *Lesothosaurus* [20] but we regard that as unlikely due to its posterior position. In these taxa, the laterosphenoid seems to correspond to the ossification of the pila antotica only, resembling the laterosphenoid *sensu* Bellairs & Kamal [82]. Whether the anterior part of the ossification was not present or has been prepared away (or if these represent juvenile individuals) is unknown, but it is likely that a reduction in the degree of ossification of the anterior braincase wall occurred in some taxa. Laterosphenoids of more derived theropods such as *Alioramus altai* [104] or *Troodon formosus* [105] are again represented by complex and extensive structures, likely encompassing multiple embryonic elements.

Irrespective of the terminology employed, our main intention here is to demonstrate that the laterosphenoids of *Euparkeria*, *Proterosuchus alexanderi*, and other basal archosaurs do not appear to be exactly the same structure as the laterosphenoid of extant crocodilians and may be more similar to that of extant birds than previously acknowledged. Also, given the delicate nature of the ossifications of the anterior braincase wall and the presence of such structures in a diapsid *incertae sedis* (*Elachistosuchus huenei* [93]), it may be that these elements were not lost in the evolutionary history of reptilians and subsequently reappeared in archosauriforms, but that it has been present throughout Diapsida, being lost only at a certain stage within Lepidosauromorpha.

### 5.2. *Euparkeria* in the context of wider diapsid braincase evolution

Since the seminal work of Gower & Weber [42], our knowledge of fossil archosauromorph braincases has increased substantially thanks to a large number of new descriptions [50,56,57,68,69,77,98,106–109], as has our understanding of the phylogenetic relationships of early and stem archosaurs [3,28,110–112]. It is thus appropriate to attempt to place the braincase of *Euparkeria* in the context of the wider archosauromorph and eureptilian radiation, and in the morphological trends seen both stemward and crownward of the taxon.

Increasing braincase height relative to anteroposterior length is identifiable as a trend in archosauromorph and diapsid braincase evolution, with Gower & Sennikov [51] first attempting to capture this change via the character of a verticalized parabasisphenoid, with the basal tubera dorsal to the basipterygoid processes. However, another degree of verticalization is found in non-archosauromorph diapsids in the contact between basioccipital and parabasisphenoid, so that the occipital condyle lies dorsal to the basal tubera. The braincase of basal eureptilians is anteroposteriorly flat in spite of a very subtle angle formed by the contact between parasphenoid and basioccipital (e.g. *Captorhinus* [54]), and the dorsoventral distance between occipital condyle and the basal tubera increases in basal diapsids (e.g. *Youngina* [50,85]). The angulation of the parabasisphenoid first appears in archosauromorphs.

In *Euparkeria*, the basal tubera are placed noticeably dorsal to the basipterygoid processes in lateral view, and the occipital condyle is in turn dorsal to the basal tubera (figures 9*c*, 10*b* and 14*e*). This contrasts with the state in the archosauromorph *Prolacerta* [46,51], and the archosauriforms *Proterosuchus fergusi* [51], and *Fugusuchus* [51], which show much more horizontal braincases. A relatively tall, verticalized parabasisphenoid is typical of crown archosaurs (e.g. *Coelophysis rhodesiensis* [113]; *Stagonolepis robertsoni* [67]), but it is also found in many archosauriforms (e.g. *Erythrosuchus* [62]; *Sarmatosuchus otschevi* [51]) and some archosauromorphs (*Trilophosaurus*—UCMP V6374; *Mesosuchus*—SAM-PK-6536). Some archosaurs, however, do show a lesser degree of angulation between basioccipital and parabasisphenoid (e.g. *Desmatosuchus spurensis* [95], *Lewisuchus admixtus* [78], *Silesaurus* [68], *Stagonolepis olenkae* [77] and proterochampsids [109]).

Another trend in diapsid and archosauromorph evolution is the increase in the participation of the basioccipital in the braincase floor. As the myelencephalon appears to correlate with the basioccipital and the parabasisphenoid with the metencephalon, this may reflect an increase in the posterior part of the hindbrain relative to the anterior part. Basal eureptilians have a parasphenoid as long as two-thirds of the anteroposterior length of the braincase (e.g. *Captorhinus* [54]). The contribution of both elements is more equal in basal diapsids (e.g. *Araeoscelis gracilis* [114]; *Youngina* [50,115]). The situation is similar in the archosauromorph *Prolacerta* [46], but the basioccipital contribution is somewhat greater in the archosauromorphs *Trilophosaurus* [70] and *Mesosuchus* (SAM-PK-6536), demonstrating that this ‘trend’ is not uniform, given that these taxa are generally placed lower on the stem than *Prolacerta* (e.g. [116]). In *Euparkeria*, and many crown taxa (e.g. *Silesaurus* [68]; *Stagonolepis robertsoni* [117]) the contribution of the parabasisphenoid to the braincase floor is limited to at most the anterior third (figure 6*c*).

**Figure 22.**
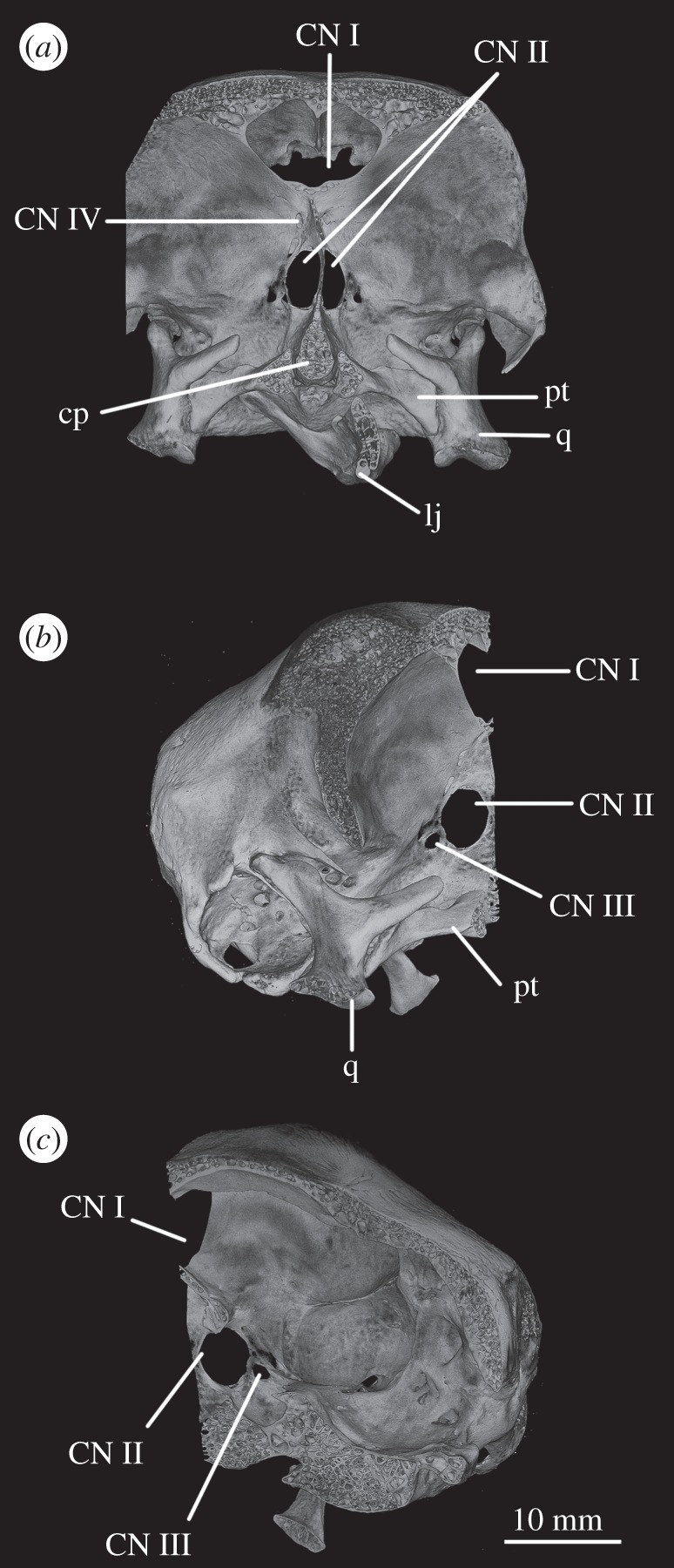
CT reconstructions of the braincase of *Meleagris gallopavo* (ZMB 1793 792) in (*a*) anterior, (*b*) right anterolateral, and (*c*) left medial views. For abbreviations, see table 1.

The contribution of the parabasisphenoid to the lateral braincase wall also tends to decrease towards the archosaur crown, as it is replaced by the anterior inferior process of the prootic. This development reflects assimilation of the embryonic pila antotica by the bone (discussed later). The parabasisphenoid forms the anteroventral border of the trigeminal notch in basal eureptilians (e.g. *Captorhinus* [54]), but in diapsids its contribution decreases due to the development of a small anterior inferior process (e.g. *Youngina* [50,115]). Participation of the parabasisphenoid disappears completely in archosauromorphs (e.g. *Trilophosaurus* [70]; *Mesosuchus*—SAM-PK-6536; *Prolacerta* [46]), including *Euparkeria*.

Further development of the prootic is exemplified by increased ossification dorsal to the foramen for CN V, by the relative positions of the trigeminal foramen and that for CN VII and by the prootic contribution to the dorsum sellae. These are all connected to assimilations of further embryonic structures by the prootic. The region of the prootic dorsal to the trigeminal notch and anterior to the otic capsule is the alar process [84], and appears to be absent, or very weakly developed, in early eureptilians (e.g. *Captorhinus* [54]). The process becomes more ossified towards the crown and in *Euparkeria*, *Fugusuchus* and *Xilousuchus* [51], and *Erythrosuchus* [62] (BPI 3893), it shows an extensive degree of ossification. The prootic may continue to expand anterodorsally in crown archosaurs, and eventually enclose the foramen of CN V entirely (e.g. *Dysalotosaurus* [56]).

The ossification of the body of the prootic is related to the otic capsule and to the prefacial and basicapsular commissures—connections of cartilage formed between the otic capsule and the basal plate [84]. Its lateral wall is characterized by the presence of the crista prootica, ventral to which the trigeminal and facial nerves exit the braincase. In basal eureptilians, the foramina for the nerves lie in the same horizontal plane, also indicated by a horizontal crest (e.g. *Captorhinus* [54]). In diapsids (e.g. *Youngina* [115]), a shift occurs in the relative positions of these foramina, with the facial foramen lying a short distance ventral to the trigeminal foramen. The crest thus curves gently in an anteroventral direction. In archosauromorphs the morphology is more varied, and some taxa show a roughly horizontal crest (e.g. *Prolacerta* [46]), while the crest of others is strongly inclined (e.g. *Mesosuchus*, SAM-PK-6536). The trigeminal and facial foramina in archosauriforms, including *Euparkeria*, are also asymmetrically positioned, albeit not to the same degree as in *Mesosuchus* (e.g. *Fugusuchus*, *Garjainia prima* [51], *Erythrosuchus* [62]). A stronger anteroventral inclination of the crista is also found in crown archosaurs (e.g. *Silesaurus* [68]; *Stagonolepis olenkae* [77]; although less so in *Xilousuchus* [51]).

The exact extent of the contribution of the prootic and parabasisphenoid to the dorsum sellae is difficult to confirm in some taxa. It is formed by the basisphenoid only in basal eureptilians (e.g. *Captorhinus* [54]), in basal diapsids (e.g. *Youngina* [50]), and at least in some archosauromorphs (e.g. *Prolacerta* [46]). The participation of the prootic in *Euparkeria* indicates assimilation of the embryonic basal plate by the prootic [84].

The hearing system also shows considerable changes along the diapsid and archosauromorph lineages. A pattern that has been extensively discussed in the evolution of the hearing system is the identification of the elements forming the border of the fenestra ovalis. The relative contributions of these bones are of particular interest in the construction of homology statements [118]. In basal eureptilians [54] and the basal diapsid *Araeoscelis gracilis* [114], the parasphenoid, basisphenoid and basioccipital contribute significantly to the ventral, anterior and posterior rims of the fenestra ovalis, respectively, with limited contribution by the prootic anterodorsally. In *Euparkeria*, the prootic and opisthotic form most of the anterior and posterior borders of the fenestra ovalis, respectively, with a small posteroventral contribution by the basioccipital on the medial side of the fenestra, and a contribution to the anterior rim by the posterodorsal process of the parabasisphenoid (figure 6*c*). In extant archosaurs, the prootic and opisthotic alone form the border of the fenestra ovalis [42], but in several extinct crown archosaurs (e.g. [56,68]) the basioccipital and parabasisphenoid are not completely excluded from the fenestra ovalis, with very restricted contributions at its ventralmost extent, while in other extinct crown taxa [95] and in erythrosuchids [62], a small basioccipital contribution at least is reported. However, this trend is not clear cut, as in the basal diapsid *Youngina*, and the archosauromorphs *Prolacerta* and *Trilophosaurus* the basioccipital appears to have been totally excluded from the fenestra ovalis by the opisthotic [46,50,70,115].

Elongation of the semicircular canals is seen in crown archosaurs in comparison to the only fossil basal diapsid for which the structure of the semicircular canals is well known—*Youngina* [50]. Strongly elongated semicircular canals are seen in flying and gliding taxa [119]. It indicates that elongation facilitates improved locomotor agility, with elongation of a particular semicircular canal corresponding to increased sensitivity in its plane of action [60,74]. The inner ear of *Euparkeria* shows relatively elongated semicircular canals with larger radii of curvature when compared with *Youngina*, especially the posterior semicircular canal. They are, however, not as strongly elongated as those of coelurosaurian theropods (especially birds [74,119,120]) and pterosaurs [119], where the anterior semicircular canal is particularly elongated. Similarly, the size of the floccular lobe, and thus the floccular fossa in which it sits, has been hypothesized to facilitate agility [121], as it emits coordination-related responses important in movements of the head and eyes [122]—although the floccular size alone may not be a good proxy for predicting the flying ability in birds [123]. An enlarged foccular lobe may be a result not only of the increase amount of floccular tissue itself but also of other parts of the vestibulocerebellum involved in postural and locomotor reflexes [123]. Expansion of these parts may be responsible for the expression of the floccular lobe through the anterior semicircular canal, and its secondary enlargement. In *Euparkeria*, the floccular fossa is much smaller than in modern birds [120], but larger than in *Youngina*, as shown not only by the increased radius of curvature of the anterior semicircular canal but also by the depth of the fossa on the medial side of the prootic. Taken together, both the form of the semicircular canals and the size of the floccular lobe may be indicative of navigation in more complex, three-dimensional environments, thus supporting a more upright, agile locomotory pattern seen in *Euparkeria* [17] than in ‘sprawling’ diapsids such as *Youngina*.

In non-saurian diapsid taxa such as *Captorhinus* and *Youngina*, there is no recess on the dorsal surface of the basioccipital/parabasisphenoid indicating that the cochlea would extend further ventrally than the limit of the braincase floor. As we interpret the distal tip of the cochlea of *Euparkeria* passing lateral to the braincase floor and medial to the bony bar connecting the ventral ramus of the opisthotic and the posterodorsal region of the parabasisphenoid, an increase in cochlear length is also evident in *Euparkeria*, indicating an improved hearing ability. Different groups of hair cells have stereocilia with varying degrees of length and stiffness, and the mass of the basilar membrane itself changes topologically. Thus, different parts of the cochlea resonate differently to the same sound frequency and the auditory epithelium is said to be tonotopically arranged along its axis [124,125]. In order to expand the hearing range, an increased number of cells are necessary, resulting in a longer cochlea. This is called mechanical tuning and is assumed to correspond to the plesiomorphic type of tonotopic discrimination in amniotes [126,127]. It has been demonstrated that cochlear length is predictive of both auditory capabilities and behaviour in extant archosaurs [128]. However, physiological tuning mechanisms such as electrical resonance became predominant during the evolutionary history of birds and turtles. In these, hair cells set up a voltage gradient via active K^+^/Ca^2+^ channels, oscillating in response to a depolarizing stimulus [126]. Although the elongation of the cochlea is also present in birds, and to a lesser extent also in crocodylians, it seems to be less important for sound discrimination than the physiological properties of the inner ear.

Because mechanical tuning is an important property of the ear of extant mammals and squamates [123,125], but not so much for birds and turtles [126], it is likely that basal archosauromorphs also relied on such mechanisms. A further, indirect indication of the importance of mechanical tuning in hearing is extensive ossification of the otic capsule. Increased ossification raises the stiffness of the system and influences frequency response by reducing energy loss due to flexion [129]. Increased ossification also promotes acoustic isolation, hindering sound conduction along routes other than those where sound detecting tissues are located. However, acoustic isolation without a compensatory mechanism for pressure relief can limit hearing capacity [73,130]. Crown group archosaurs developed a specialized pressure-relief window, a fenestra pseudorotunda, in which the metotic foramen becomes subdivided into anterior and posterior regions [42]. The compensatory mechanism of the fenestra pseudorotunda can, to a lesser extent, be carried out by the undivided metotic foramen [84]. The medial wall of the otic capsule of most eureptilians is extensively unossified, but the metotic foramen becomes increasingly enlarged. The metotic foramen of *Euparkeria* is even more enlarged than that of *Captorhinus* [54], *Youngina* [115] or *Prolacerta* [46], and there is further differentiation of a pressure-relief region ventrally.

Some of the changes seen in the lineage leading to *Euparkeria*, and subsequently within the archosaur crown, relate to the changing ecomorphology of the taxa. A general increase in ossification of the braincase may be indicative of development of the forebrain and improvement of terrestrial hearing. Furthermore, increased length of the semicircular canals and increased size of the floccular lobe are probably indicative of the change from a sprawling to upright gait. The changes witnessed along the lineage leading to *Euparkeria* and beyond can thus be framed as part of the broader terrestrial amniote pattern of increasing adaptation to terrestriality, including locomotion and hearing, and of development of increased cognitive abilities.

## 6. Conclusion

For the first time, a complete description of the braincase of *Euparkeria* is undertaken based on all available material. We were able confirm and correct several details of descriptions published previously. For instance, we confirm the presence of a laterosphenoid in the anterior braincase region of *Euparkeria*, and find that the element may not be fully homologous with that present in extant crocodylians, with the crocodilian condition being less ossified. We also homologize the ATR of *Eupakeria* with that of other basal archosaurs. The elongation of the semicircular canals (the anterior in particular) and the enlargement of the floccular fossa may correspond to development of a more upright quadrupedal posture and a more active lifestyle in *Euparkeria* than in basal diapsids and taxa further down the archosaur stem. The enlargement of the fenestra ovalis and of the metotic foramen, together with the regionalization of the latter and the elongation of the cochlea, are considered to be related to extension of the hearing range and improvements in the impedance matching functions of the inner ear, pointing to further development of a sense of hearing more adapted to terrestrial environments.

## Supplementary Material

Figure S1

## Supplementary Material

Figure S2

## Supplementary Material

Figure S3

## Supplementary Material

SUPPLEMENTARY FIGURES CAPTIONS

## Data accessibility

Given the large size of the individual three-dimensional models, all data generated by computed tomography scanning of the materials are deposited in the virtual database of the Museum für Naturkunde Berlin, and are available upon request.

## References

[1] Jetz W, Thomas GH, Joy JB, Hartmann K, Mooers AO 2012 The global diversity of birds in space and time. Nature 491, 444–448. (doi:10.1038/nature11631) 23123857 10.1038/nature11631

[2] Irmis RB, Nesbitt SJ, Padian K, Smith ND, Turner AH, Woody D, Downs A 2007 A Late Triassic dinosauromorph assemblage from New Mexico and the rise of the dinosaurs. Science 317, 358–361. (doi:10.1126/science.1143325) 17641198 10.1126/science.1143325

[3] Brusatte SL, Benton MJ, Desojo JB, Langer MC 2010 The higher-level phylogeny of Archosauria (Tetrapoda: Diapsida). J. Syst. Palaeontol. 8, 3–47. (doi:10.1080/14772010903537732)

[4] Brusatte SL, Benton MJ, Lloyd GT, Ruta M, Wang SC 2011 Macroevolutionary patterns in the evolutionary radiation of archosaurs (Tetrapoda: Diapsida). Earth Environ. Sci. Trans. R. Soc. Edinb. 101, 367–382. (doi:10.1017/S1755691011020056)

[5] Charig AJ 1980 Differentiation of lineages among Mesozoic tetrapods. Mém. Soc. Géol. Fr. 139, 207–210.

[6] Benton MJ 1984 Dinosaur success in the Triassic: a noncompetitive ecological model. Q. Rev. Biol. 58, 29–55. (doi:10.1086/413056)

[7] Tucker ME, Benton MJ 1982 Triassic environments, climates and reptile evolution. Palaeogeogr. Palaeoclimatol. *Palaeoecol.* 40, 361–379. (doi:10.1016/0031-0182(82)90034-7)

[8] Benton MJ 1984 Dinosaurs’ lucky break. Nat. Hist. 93, 54–59.

[9] Benton MJ 1984 Rauisuchians and the success of dinosaurs. Nature 310, 101 (doi:10.1038/310101a0)

[10] Sues H-D, Fraser NC 2013 Triassic life on land: the great transition. New York, NY: Columbia University Press.

[11] Sahney S, Benton MJ 2008 Recovery from the most profound mass extinction of all time. Proc. R. Soc. B 275, 759–765. (doi:10.1098/rspb.2007.1370) 10.1098/rspb.2007.1370 PMC2596898 18198148

[12] Brusatte SL, Benton MJ, Ruta M, Lloyd GT 2008 Superiority, competition, and opportunism in the evolutionary radiation of the dinosaurs. Science 321, 1485–1488. (doi:10.1126/science.1161833) 18787166 10.1126/science.1161833

[13] Sookias RB, Benson RBJ, Butler RJ 2012 Biology, not environment, drives major patterns in maximum tetrapod body size through time. Biol. Lett. 8, 674–677. (doi:10.1098/rsbl.2012.0060) 22513278 10.1098/rsbl.2012.0060 PMC3391459

[14] Ewer RF 1965 The anatomy of the thecodont reptile *Euparkeria capensis* Broom. Phil. Trans. R. Soc. Lond. B 248, 379–435. (doi:10.1098/rstb.1965.0003)

[15] Hancox PJ, Shishkin MA, Rubidge BS, Kitching JW 1995 A threefold subdivision of the *Cynognathus* Assemblage Zone (Beaufort Group, South Africa) and its palaeogeographical implications. S. Afr. J. Sci. 91, 143–144.

[16] Hancox PJ 2000 The continental Triassic of South Africa. Zbl. Geol. Paläontol. I 1998, 1285–1324.

[17] Sookias RB, Butler RJ 2013 Euparkeriidae. In Anatomy, phylogeny and palaeobiology of early archosaurs and their kin (eds Nesbitt SJ, Desojo JB, Irmis RB), pp. 35–48. Special Publication 379. London, UK: Geological Society of London.

[18] Benton MJ, Clark JM 1988 Archosaur phylogeny and the relationships of the Crocodylia. In The phylogeny and classification of tetrapods, volume 1: amphibians, reptiles, birds (ed. Benton MJ), pp. 289–332. Oxford, UK: Clarendon Press.

[19] Sereno PC, Arcucci AB 1990 The monophyly of crurotarsal archosaurs and the origin of bird and crocodile ankle joints. N. Jb. Geol. Paläontol. Abh. 180, 21–52.

[20] Sereno PC 1991 Basal archosaurs: phylogenetic relationships and functional implications. Mem. Soc. Vert. Paleontol. 2, 1–53. (doi:10.2307/3889336)

[21] Parrish JM 1993 Phylogeny of the Crocodylotarsi, with reference to archosaurian and crurotarsan monophyly. J. Vert. Paleontol. 13, 287–308. (doi:10.1080/02724634.1993.10011511)

[22] Juul L 1994 The phylogeny of basal archosaurs. Palaeontol. Afr. 31, 1–38.

[23] Bennett SC 1996 The phylogenetic position of the Pterosauria within the Archosauromorpha. Zool. J. Linn. Soc. 118, 261–308. (doi:10.1080/08912963.2012.725727)

[24] Benton MJ 1999 *Sceromochlus taylori* and the origin of dinosaurs and pterosaurs. Phil. Trans. R. Soc. Lond. B 354, 1423–1446. (doi:10.1098/rstb.1999.0489)

[25] Parker WG, Barton BJ 2008 New information on the Upper Triassic archosauriform *Vancleavea campi* based on new material from the Chinle Formation of Arizona. Palaeontol. Electron. 11, 14A.

[26] Nesbitt SJ, Stocker MR, Small BJ, Downs A 2009 The osteology and relationships of *Vancleavea campi* (Reptilia: Archosauriformes). Zool. J. Linn. Soc. 157, 814–864. (doi:10.1111/j.1096-3642.2009.00530.x)

[27] Ezcurra MD, Lecuona A, Martinelli A 2010 A new basal archosauriform diapsid from the Lower Triassic of Argentina. J. Vert. Paleontol. 30, 1433–1450. (doi:10.1080/02724634.2010.501446)

[28] Nesbitt SJ 2011 The early evolution of archosaurs: relationships and the origin of major clades. Bull. Am. Mus. Nat. Hist. 352, 1–292. (doi:10.1206/352.1)

[29] Perry SF 1992 Gas exchange strategies in reptiles and the origin of the avian lung. In Physiological adaptations in vertebrates: respiration, circulation, and metabolism (eds Wood SC, Weber RE, Hargens AR, Millard RW), pp. 149–167. New York, NY: Marcel Dekker.

[30] Carrier DR, Farmer CG 2000 The evolution of pelvic aspiration in archosaurs. Paleobiology 26, 271–293. (doi:10.1666/0094-8373(2000)026<0271:TEOPAI>2.0.CO;2)

[31] Hutchinson JR 2001 The evolution of femoral osteology and soft tissues on the line to extant birds (Neornithes). Zool. J. Linn. Soc. 131, 169–197. (doi: 10.1111/j.1096-3642.2001.tb01314.x)

[32] Hutchinson JR 2001 The evolution of pelvic osteology and soft tissues on the line to extant birds (Neornithes). Zool. J. Linn. Soc. 131, 123–168. (doi:10.1111/j.1096-3642.2001.tb01313.x)

[33] Marugán-Lobón J, Buscalioni AD 2003 Disparity and geometry of the skull in Archosauria (Reptilia: Diapsida). Zool. J. Linn. Soc. 80, 67–88. (doi:10.1046/j.1095-8312.2003.00219.x)

[34] Nesbitt SJ 2003 *Arizonasaurus* and its implications for archosaur divergence. Proc. R. Soc. Lond. B 270, S234–S237. (doi:10.1098/rsbl.2003.0066) 10.1098/rsbl.2003.0066 PMC1809943 14667392

[35] Rauhut OWM 2003 The interrelationships and evolution of basal theropod dinosaurs. Spec. Pap. Palaeontol. 69, 1–213.

[36] Seymour RS, Bennett-Stamper CL, Johnston SD, Carrier DR, Grigg GC 2004 Evidence for endothermic ancestors of crocodiles at the stem of archosaur evolution. Physiol. Biochem. Zool. 77, 1051–1067. (doi:10.1086/422766) 15674775 10.1086/422766

[37] de Ricqles A, Padian K, Knoll F, Horner JR 2008 On the origin of high growth rates in archosaurs and their ancient relatives: complementary histological studies on Triassic archosauriforms and the problem of a ‘phylogenetic signal’ in bone histology. Ann. Paleontol. 94, 57–76. (doi:10.1016/j.annpal.2008.03.002)

[38] Sullivan C 2010 The role of the calcaneal ‘heel’ as a propulsive lever in basal archosaurs and extant monitor lizards. J. Vert. Paleontol. 30, 1422–1432. (doi:10.1080/02724634.2010.501450)

[39] Maidment SCR, Barrett PM 2011 The locomotor musculature of basal ornithischian dinosaurs. J. Vert. Paleontol. 31, 1265–1291. (doi:10.1080/039.031.0609)

[40] Butler RJ, Barrett PM, Gower DJ 2012 Reassessment of the evidence for postcranial skeletal pneumaticity in Triassic archosaurs, and the early evolution of the avian respiratory system. PLoS ONE 7, e34094 (doi:10.1371/journal.pone.0034094) 22470520 10.1371/journal.pone.0034094 PMC3314707

[41] Foth C, Rauhut OWM 2013 Macroevolutionary and morphofunctional patterns in theropod skulls: a morphometric approach. Acta Palaeontol. Pol. 58, 1–16. (doi:10.4202/app.2011.0145)

[42] Gower DJ, Weber E 1998 The braincase of *Euparkeria*, and the evolutionary relationships of birds and crocodilians. Biol. Rev. 73, 367–411. (doi:10.1111/j.1469-185X.1998.tb00177.x)

[43] Gauthier JA, Nesbitt SJ, Schachner ER, Bever GS, Joyce WG 2011 The bipedal stem crocodilian *Poposaurus gracilis*: inferring function in fossils and innovation in archosaur locomotion. Bull. Peabody Mus. Nat. Hist. 52, 107–126. (doi:10.3374/014.052.0102)

[44] Kubo T, Kubo MO 2012 Associated evolution of bipedality and cursoriality among Triassic archosaurs: a phylogenetically controlled evaluation. Paleobiology 38, 474–485. (doi:10.1666/11015.1)

[45] Cruickshank ARI 1970 Early thecodont braincases. Proc. Intern. Gondwana Symp. 2, 683–685.

[46] Evans SE 1986 The braincase of *Prolacerta broomi* (Reptilia, Triassic). N. Jb. Geol. Paläontol. Abh. 173, 181–200.

[47] Welman J 1995 Euparkeria and the origin of birds. S. Afr. J. Sci. 91, 533–537.

[48] Gauthier JA 1986 Saurischian monophyly and the origin of birds. Mem. Cal. Acad. Sci. 8, 1–55.

[49] Sookias RB, Sennikov AG, Gower DJ, Butler RJ 2014 The monophyly of Euparkeriidae (Reptilia: Archosauriformes) and the origins of crown Archosauria: a revision of *Dorosuchus neoetus* from the Middle Triassic of Russia. Palaeontology 57, 1177–1202. (doi:10.5061/dryad.n525j)

[50] Gardner NM, Holliday CM, O'Keefe FR 2010 The braincase of *Youngina capensis* (Reptilia, Diapsida): new insights from high-resolution CT scanning of the holotype. Palaeontol. Electron. 13, 16.

[51] Gower DJ, Sennikov AG 1996 Morphology and phylogenetic informativeness of early archosaur braincases. Palaeontology 39, 883–906.

[52] Witmer LM 1997 The evolution of the antorbital cavity of archosaurs: a study in soft-tissue reconstruction in the fossil record with an analysis of the function of pneumaticity. Mem. Soc. Vert. Paleontol. 3, 1–73. (doi:10.1080/02724634.1997.10011027)

[53] Evans SE 1980 The skull of a new eosuchian reptile from the Lower Jurassic of South Wales. Zool. J. Linn. Soc. 70, 203–264. (doi:10.1111/j.1096-3642.1980.tb00852.x)

[54] Heaton MJ 1979 Cranial anatomy of primitive captorhinid reptiles from the late Pennsylvanian and Early Permian of Oklahoma and Texas. Oklah. Geol. Surv. Bull. 127, 1–81.

[55] Oelrich TM 1956 The anatomy of the head of *Ctenosaura pectinata* (Iguanidae). Misc. Publ. Mus. Zool. Univ. Mich. 94, 11–22.

[56] Sobral G, Hipsley CA, Müller J 2012 Braincase redescription of *Dysalotosaurus lettowvorbecki* (Dinosauria, Ornithopoda) based on computed tomography. J. Vert. Paleontol. 32, 1090–1102. (doi:10.1080/02724634.2012.693554)

[57] Gower DJ 2002 Braincase evolution in suchian archosaurs (Reptilia: Diapsida): evidence from the rauisuchian *Batrachotomus kupferzellensis*. Zool. J. Linn. Soc. 136, 49–76. (doi:10.1046/j.1096-3642.2002.00025.x)

[58] Bruner HL 1907 On the cephalic veins and sinuses of reptiles, with description of a mechanism for raising the venous blood-pressure in the head. Am. J. Anat. 7, 1–117. (doi:10.1002/aja.1000070102)

[59] Dendy A 1909 The intracranial vascular system of *Sphenodon*. Phil. Trans. R. Soc. B 200, 403–426. (doi:10.1098/rspb.1909.0025)

[60] Sampson SD, Witmer LM 2007 Craniofacial anatomy of *Majungasaurus crenatissimus* (Theropoda: Abelisauridae) from the Late Cretaceous of Madagascar. Mem. Soc. Vert. Paleontol. 8, 32–102. (doi:10.1671/0272-4634(2007)27[32:CAOMCT]2.0.CO;2)

[61] Borsuk-Białynicka M, Evans SE 2009 Cranial and mandibular osteology of the Early Triassic archosauriform *Osmolskina czatkowicensis* from Poland. Palaeontol. Pol. 65, 235–281.

[62] Gower DJ 1997 The braincase of the early archosaurian reptile *Erythrosuchus africanus*. Proc. Zool. Soc. Lond. 242, 557–576. (doi:10.1111/j.1469-7998.1997.tb03855.x)

[63] Clark JM, Welman J, Gauthier JA, Parrish MJ 1993 The laterosphenoid of bone of early archosauriforms. J. Vert. Paleontol. 13, 48–57. (doi:10.1080/02724634.1993.10011487)

[64] Holliday CM, Witmer LM 2009 The epipterygoid of crocodyliforms and its significance in the evolution of the orbitotemporal region of eusuchians. J. Vert. Paleontol. 29, 715–733. (doi:10.1671/039.029.0330)

[65] Miyashita T, Arbour VM, Witmer LM, Currie PJ 2011 The internal cranial morphology of an armoured dinosaur *Euoplocephalus* corroborated by X-ray computed tomographic reconstruction. J. Anat. 219, 661–675. (doi:10.1111/j.1469-7580.2011.01427.x) 21954840 10.1111/j.1469-7580.2011.01427.x PMC3237876

[66] Willard WA 1915 The cranial nerves of *Anolis carolinensis*. Bull. Mus. Comp. Zool. Harv. Col. 59, 18–116.

[67] Walker AD 1990 A revision of *Sphenosuchus acutus* Haughton, crocodylomorph reptile from the Elliot Formation (Late Triassic or Early Jurassic) of South Africa. Phil. Trans. R. Soc. Lond. B 330, 1–120. (doi:10.1098/rstb.1990.0185)

[68] Dzik J 2003 A beaked herbivorous archosaur with dinosaur affinities from the early Late Triassic of Poland. J. Vert. Paleontol. 23, 556–574. (doi:10.1671/A1097)

[69] Gower DJ, Nesbitt SJ 2006 The braincase of *Arizonasaurus babbitti*—further evidence of the non-monophyly of Rauisuchia. J. Vert. Paleontol. 26, 79–87. (doi:10.1671/0272-4634(2006)26[79:TBOABE]2.0.CO;2)

[70] Gregory JT 1945 Osteology and relationships of *Trilophosaurus*. Univ. Tex. Publ. 4401, 273–359.

[71] de Beer GR, Barrington EJW 1934 The segmentation and chondrification of the skull of the duck. Phil. Trans. R. Soc. Lond. B 223, 411–467. (doi:10.1098/rstb.1934.0009)

[72] Witmer LM 1990 The craniofacial air sac system of Mesozoic birds (Aves). Zool. J. Linn. Soc. 100, 327–378. (doi:10.1111/j.1096-3642.1990.tb01865.x)

[73] Wever EG 1978 The reptile ear: its structure and function. Princeton, NJ: Princeton University Press.

[74] Witmer LM, Ridgely RC, Dufeau DL, Semones MC 2008 Using CT to peer into the past: 3D visualization of the brain and ear regions of birds, crocodiles, and nonavian dinosaurs. In Anatomical imaging: towards a new morphology (eds Endo H, Frey R), pp. 67–88. Tokyo, Japan: Springer.

[75] Witmer LM, Ridgely RC 2009 New insights into the brain, braincase, and ear region of tyrannosaurs, with implications for sensory organization and behavior. Anat. Rec. 292, 1266–1296. (doi:10.1002/ar.20983) 10.1002/ar.20983 19711459

[76] Butler RJ, Sullivan C, Ezcurra MD, Liu J, Lecuona A, Sookias RB 2014 New clade of enigmatic early archosaurs yields insights into early pseudosuchian phylogeny and the biogeography of the archosaur radiation. BMC Evol. Biol. 14, 128 (doi:10.1186/1471-2148-14-128) 24916124 10.1186/1471-2148-14-128 PMC4061117

[77] Sulej T 2010 The skull of an early Late Triassic aetosaur and the evolution of the stagonolepidid archosaurian reptiles. Zool. J. Linn. Soc. 158, 860–881. (doi:10.1111/j.1096-3642.2009.00566.x)

[78] Bittencourt JS, Arcucci AB, Marsicano CA, Langer MC 2014 Osteology of the Middle Triassic archosaur *Lewisuchus admixtus* Romer (Chañares Formation, Argentina), its inclusivity, and relationships among early dinosauromorphs. J. Syst. Paleontol. 13, 1–31. (doi:10.1080/14772019.2013.878758)

[79] Mastrantonio BM, Schultz CL, Desojo JB, Garcia JB 2013 The braincase of *Prestosuchus chiniquensis* (Archosauria: Suchia). In Anatomy, phylogeny and palaeobiology of early archosaurs and their kin (eds Nesbitt SJ, Desojo JB, Irmis RB), pp. 425–440. Special Publication 379. London, UK: Geological Society of London.

[80] Chatterjee S 1993 *Shuvosaurus*, a new theropod. Nat. Geo. Res. Expl. 9, 274–285.

[81] Lehane J 2005 Anatomy and relationships of *Shuvosaurus*, a basal theropod from the Triassic of Texas. MSc Thesis, Texas Tech University.

[82] Bellairs A, Kamal AM 1981 The chondrocranium and the development of the skull in recent reptiles. In Biology of the Reptilia, volume 11, morphology F (eds Gans C, Parsons TS), pp. 1–283. New York, NY: Academic Press.

[83] de Beer GR 1937 The development of the vertebrate skull. Oxford, UK: Oxford University Press.

[84] Evans SE 2008 The skull of lizards and tuatara. In Biology of the Reptilia: the skull of Lepidosauria, volume 20, morphology H (eds Gans C, Gaunt AS, Adler K), pp. 1–347. Salt Lake City, UT: Society for the Study of Amphibians and Reptiles.

[85] Bhullar B-AS, Bever GS 2009 An archosaur-like laterosphenoid in early turtles (Reptilia: Pantestudines). Breviora 518, 1–11. (doi:10.3099/0006-9698-518.1.1)

[86] Case EC 1911 A revision of the Cotylosauria of North America. Carn. Inst. Wash. Publ. 145, 122 (doi:10.5962/bhl.title.45604)

[87] Fracasso MA 1987 Braincase of *Limnoscelis paludi* Williston. Postilla 201, 1–22.

[88] Modesto SP, Reisz RR 2008 New material of *Colobomycter pholeter*, a small parareptile from the Lower Permian of Oklahoma. J. Vert. Paleontol. 28, 677–684. (doi:10.1671/0272-4634(2008)28[677:NMOCPA]2.0.CO;2)

[89] Modesto SP, Scott DM, Berman DS, Müller J, Reisz RR 2007 The skull and the palaeoecological significance of *Labidosaurus hamatus*, a captorhinid reptile from the Lower Permian of Texas. Zool. J. Linn. Soc. 149, 237–262. (doi:10.1111/j.1096-3642.2007.00242.x)

[90] De Braga M, Rieppel O 1997 Reptile phylogeny and the interrelationships of turtles. Zool. J. Linn. Soc. 120, 281–354. (doi:10.1111/j.1096-3642.1997.tb01280.x)

[91] Pardo JD, Szostakiwskyj M, Anderson JS 2015 Cranial morphology of the brachystelechid ‘microsaur’ *Quasicaecilia texana* Carroll provides new insights into the diversity and evolution of braincase morphology in recumbirostran ‘microsaurs’. PLoS ONE 10, e0130359 (doi:10.1371/journal.pone.0130359) 26107260 10.1371/journal.pone.0130359 PMC4479878

[92] Romer AS 1956 Osteology of the reptiles. Chicago, IL: University of Chicago Press.

[93] Sobral G, Sues H-D, Müller J 2015 Anatomy of the enigmatic reptile *Elachistosuchus huenei* Janensch, 1949 (Reptilia: Diapsida) from the Upper Triassic of Germany and its relevance for the origin of Sauria. PLoS ONE 10, e0135114 (doi:10.1371/journal.pone.0135114) 26352985 10.1371/journal.pone.0135114 PMC4564268

[94] Olson EC 1936 Notes on the skull of *Youngina capensis* Broom. J. Geol. 44, 523–533. (doi:10.1086/624447)

[95] Small BS 2002 Cranial anatomy of *Desmatosuchus haplocerus* (Reptilia: Archosauria: Stagonolepidae). Zool. J. Linn. Soc. 136, 97–111. (doi:10.1046/j.1096-3642.2002.00028.x)

[96] Baumel JJ 1993 Handbook of avian anatomy: nomina anatomica avium. Cambridge, UK: Nuttall Ornithological Club.

[97] Livezey BC, Zusi RL 2006 Higher-order phylogeny of modern birds (Theropoda, Aves: Neornithes) based on comparative anatomy. I. Methods and characters. Bull. Carn. Mus. Nat. Hist. 37, 1–544. (doi:10.1111/j.1096-3642.2006.00293.x) 10.1111/j.1096-3642.2006.00293.x PMC2517308 18784798

[98] Balanoff AM, Bever GS, Ikejiri T 2010 The braincase of *Apatosaurus* (Dinosauria: Sauropoda) based on the computed tomography of a new specimen with comments on variation and evolution in sauropod neuroanatomy. Am. Mus. Nov. 3677, 1–29. (doi:10.1206/591.1)

[99] Janensch W 1935 Die Schädel der Sauropoden *Brachiosaurus*, *Barosaurus* und *Dicraeosaurus* aus der Tendaguru-Schichten Deutsch-Ostafrikas. Palaeontogr. Suppl. 7, 147–298.

[100] Madsen JH, McIntosh JS, Berman DS 1995 Skull and atlas-axis complex of the Upper Jurassic sauropod *Camarasaurus* Cope (Reptilia: Saurischia). Bull. Carn. Mus. Nat. Hist. 31, 1–43.

[101] Norman DB, Crompton AW, Butler RJ, Porro LB, Charig AJ 2011 The Lower Jurassic ornithischian dinosaur *Heterodontosaurus tucki* Crompton & Charig, 1962: cranial anatomy, functional morphology, taxonomy, and relationships. Zool. J. Linn. Soc. 163, 182–276. (doi:10.1111/j.1096-3642.2011.00697.x)

[102] Boyd CA 2014 The cranial anatomy of the neornithischian dinosaur *Thescelosaurus neglectus*. PeerJ 2, e669 (doi:10.7717/peerj.669) 25405076 10.7717/peerj.669 PMC4232843

[103] Galton PM 1974 The ornithischian dinosaur Hypsilophodon from the Wealden of the Isle of Wight. Bull. Br. Mus. Nat. Hist. Geol. 25, 1–152.

[104] Bever GS, Brusatte SL, Carr TD, Xu X, Balanoff AM, Norell MA 2013 The braincase anatomy of the Late Cretaceous dinosaur *Alioramus* (Theropoda: Tyrannosauroidea). Bull. Am. Mus. Nat. Hist. 376, 1–72. (doi:10.1206/810.1)

[105] Currie PJ, Zhao X-J 1993 A new troodontid (Dinosauria, Theropoda) braincase from the Dinosaur Park Formation (Campanian) of Alberta. Can. J. Earth Sci. 30, 2231–2247. (doi:10.1139/e93-194)

[106] Carabajal AP, Coria RA, Chiappe LM 2008 An incomplete Upper Cretaceous titanosaur (Sauropoda) braincase: new insights on the dinosaurian inner ear and endocranium. Cretaceous Res. 29, 643–648. (doi:10.1016/j.cretres.2008.01.011)

[107] García RA, Carabajal AP, Salgado L 2008 A new titanosaurian braincase from the Allen Formation (Campanian-Maastrichtian), Río Negro Province, Patagonia, Argentina. Geobios 41, 625–633. (doi:10.1016/j.geobios.2007.11.005)

[108] Fernández MS, Carabajal AP, Gaspirini Z, Díaz GC 2011 A metriorhynchid crocodyliform braincase from northern Chile. J. Vert. Paleontol. 32, 269–377. (doi:10.1080/02724634.2011.550361)

[109] Trotteyn MJ, Haro JA 2011 The braincase of a specimen of *Proterochampsa* Reig (Archosauriformes: Proterochampsidae) from the Late Triassic of Argentina. Paläontol. Z. 85, 1–17. (doi:10.1007/s12542-010-0068-7)

[110] Benton MJ 2004 Origin and relationships of Dinosauria. In Dinosauria, 2nd edn (eds Weishampel DB, Dobson P, Osmólska H), pp. 7–24. Berkeley, CA: University of California Press.

[111] Nesbitt SJ, Norell MA 2006 Extreme convergence in the body plans of an early suchian (Archosauria) and ornithomimid dinosaurs (Theropoda). Proc. R. Soc. B 273, 1045–1048. (doi:10.1098/rspb.2005.3426) 10.1098/rspb.2005.3426 PMC1560254 16600879

[112] Nesbitt SJ 2007 The anatomy of *Effigia okeeffeae* (Archosauria, Suchia), theropod convergence, and the distribution of related taxa. Bull. Am. Mus. Nat. Hist. 302, 1–84. (doi:10.1206/0003-0090(2007)302[1:TAOEOA]2.0.CO;2)

[113] Raath MA 1969 A new coelurosaurian dinosaur from the Forest Sandstone of Rhodesia. Arnoldia 28, 1–25.

[114] Vaughn PP 1955 The Permian reptile *Araeoscelis* re-studied. Bull. Mus. Comp. Zool. Harv. Col. 113, 305–467.

[115] Evans SE 1987 The braincase of *Youngina capensis* (Reptilia: Diapsida; Permian). N. Jb. Geol. Paläontol. Monatsh. 1987, 293.

[116] Dilkes DW 1998 The Early Triassic rhynchosaur *Mesosuchus browni* and the interrelationships of the basal archosauromorph reptiles. Phil. Trans. R. Soc. Lond. B 353, 501–541. (doi:10.1098/rstb.1998.0225)

[117] Gower DJ, Walker AD 2002 New data on the braincase of the aetosaurian archosaur (Reptilia: Diapsida) *Stagonolepis robertsoni* Agassiz. Zool. J. Linn. Soc. 136, 7–23. (doi:10.1046/j.1096-3642.2002.00023.x)

[118] Clack JA, Allin E 2004 The evolution of single- and multiple-ossicle ears in fishes and tetrapods. In Evolution of the vertebrate auditory system (eds Manley GA, Popper A, Fay RR), pp. 238–263. New York, NY: Springer.

[119] Witmer LM, Chatterjee S, Franzosa J, Rowe T 2003 Neuroanatomy of flying reptiles and implications for flight, posture and behaviour. Nature 425, 950–953. (doi:10.1038/nature02048) 14586467 10.1038/nature02048

[120] Balanoff AM, Bever GS, Norell MA 2014 Reconsidering the avian nature of the oviraptorosaur brain (Dinosauria: Theropoda). PLoS ONE 9, e113559 (doi:10.1371/journal.pone.0113559) 25494183 10.1371/journal.pone.0113559 PMC4262302

[121] Cox PG, Jeffery N 2010 Semicircular canals and agility: the influence of size and shape measures. J. Anat. 216, 37–47. (doi:10.1111/j.1469-7580.2009.01172.x) 20002227 10.1111/j.1469-7580.2009.01172.x PMC2807974

[122] Kardong K 2001 Vertebrates: comparative anatomy, function, evolution. New York, NY: McGraw-Hill.

[123] Walsh SA, Iwaniuk AN, Knoll MA, Bourdon E, Barrett PM, Milner AC, Nudds R, Abel RL, Dello Sterpaio P 2013 Avian cerebellar floccular fossa size is not a proxy for flying ability in birds. PLoS ONE 8, e67176 (doi:10.1371/journal.pone.0067176) 23825638 10.1371/journal.pone.0067176 PMC3692442

[124] Manley GA, Clack JA 2004 An outline of the evolution of vertebrate hearing organs. In Evolution of the vertebrate auditory system (eds Manley GA, Popper A, Fay RR), pp. 1–26. New York, NY: Springer.

[125] Lewis ER, Leverenz EL, Koyama H 1982 The tonotopic organization of the bullfrog amphibian papilla, an auditory organ lacking a basilar membrane. J. Comp. Physiol. 145, 437–445. (doi:10.1007/BF00612809)

[126] Manley GA, Köppl C 2008 What have lizard ears taught us about auditory physiology? Hear. Res. 238, 3–11. (doi:10.1016/j.heares.2007.09.011) 17983712 10.1016/j.heares.2007.09.011

[127] Mann ZF, Kelley MW 2011 Development of tonotopy in the auditory periphery. Hear. Res. 276, 2–15. (doi:10.1016/j.heares.2011.01.011) 21276841 10.1016/j.heares.2011.01.011

[128] Walsh SA, Barrett PM, Milner AC, Manley G, Witmer LM 2009 Inner ear anatomy is a proxy for deducing auditory capability and behaviour in reptiles and birds. Proc. R. Soc. B 276, 1355–1360. (doi:10.1098/rspb.2008.1390) 10.1098/rspb.2008.1390 PMC2660952 19141427

[129] Lombard RE, Hetherington TE 1993 Structural basis of hearing and sound transmission. In The skull, vol. 3 (eds Hanken J, Hall BK), pp. 241–302. Chicago, IL: University of Chicago Press.

[130] Manley GA 1990 Peripheral hearing mechanisms in reptiles and avialans. Berlin, Germany: Springer.

