# Supplementary figures and images for: New information on the braincase and inner ear of Euparkeria capensis Broom: implications for diapsid and archosaur evolution
Source: R Soc Open Sci. 2016 Jul 13;3(7):160072. doi: 10.1098/rsos.160072 (PMC4968458; doi:10.1098/rsos.160072)

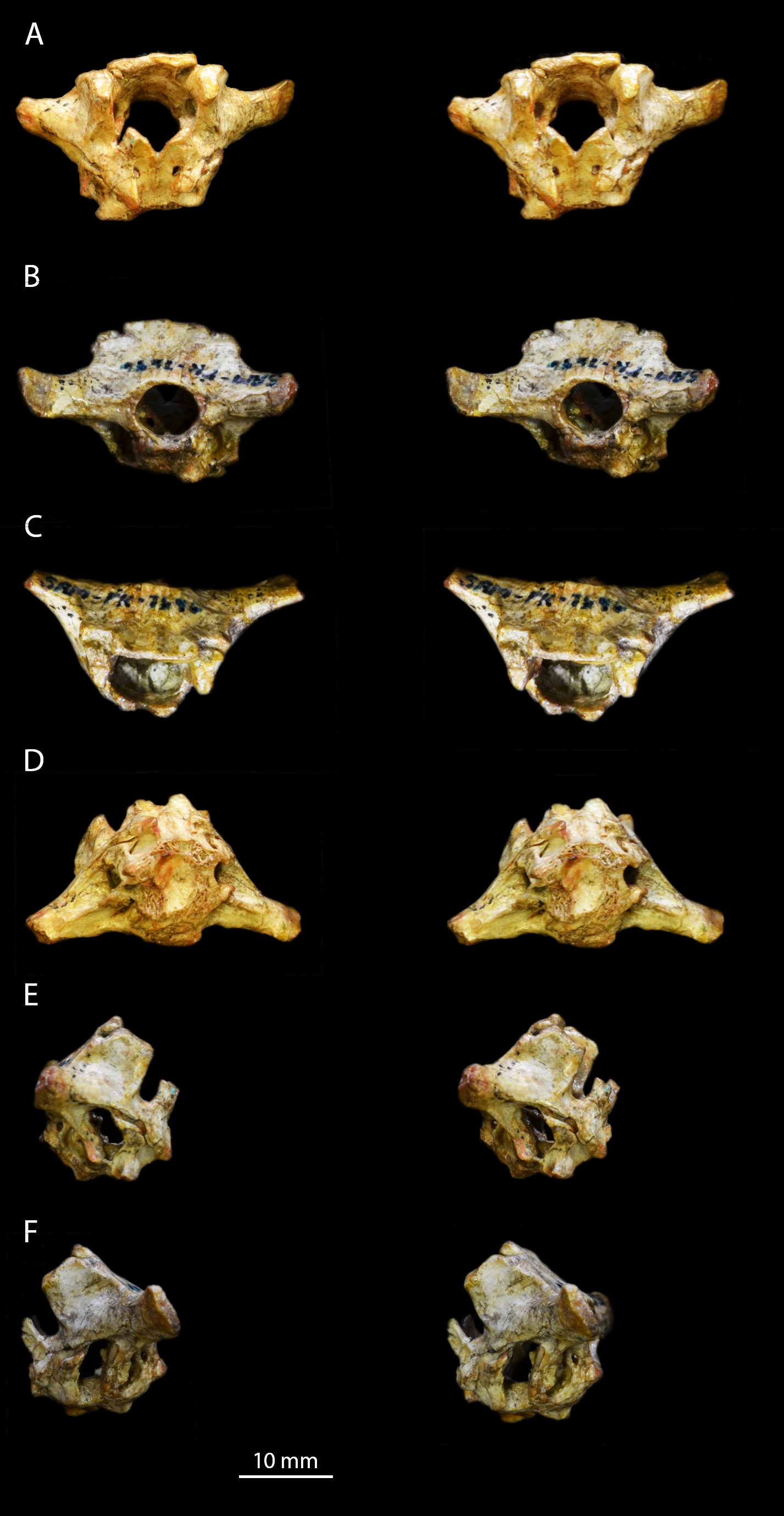

Supplement: Figure S1 [file rsos160072supp1.tif]

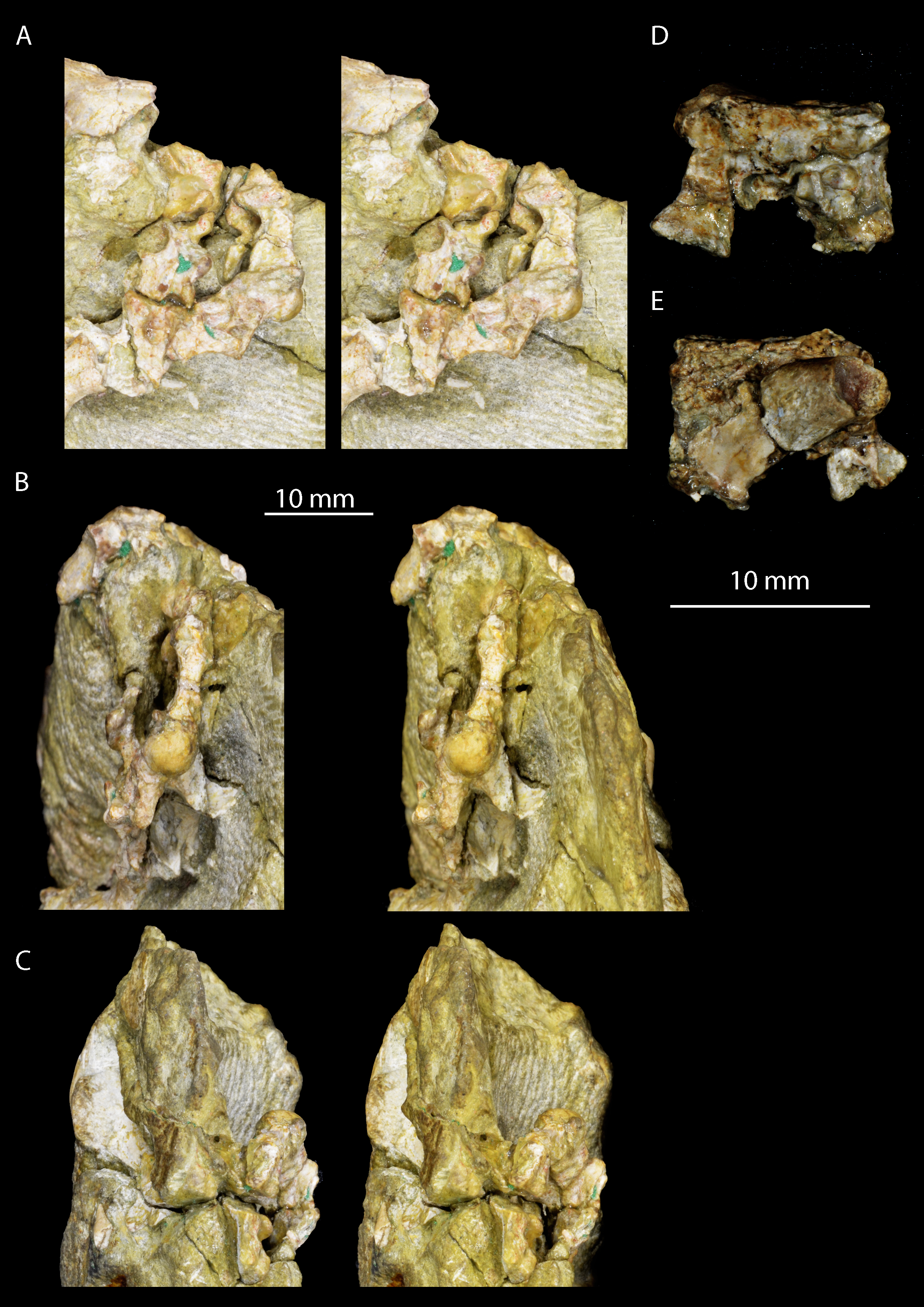

Supplement: Figure S2 [file rsos160072supp2.tif]

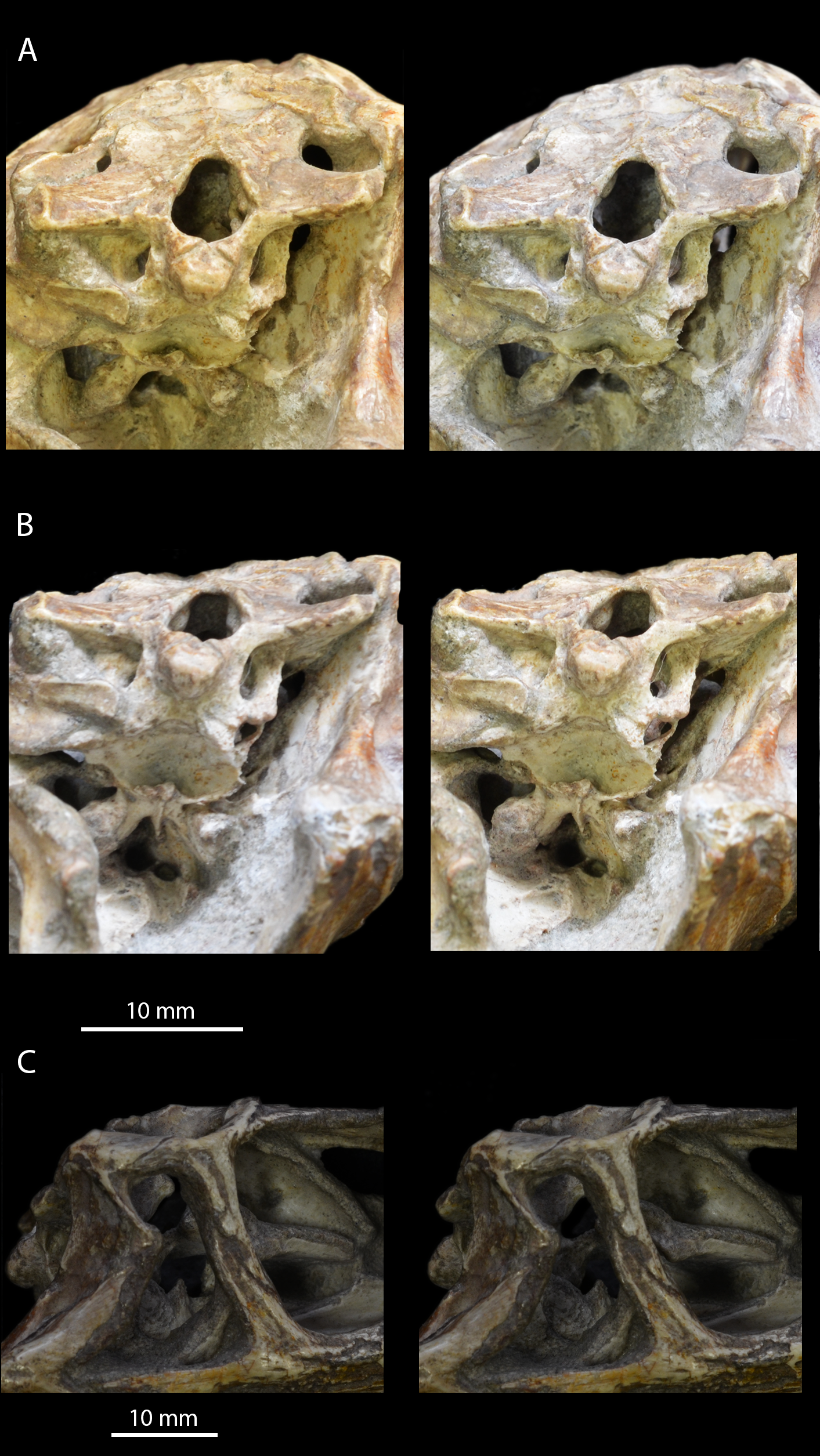

Supplement: Figure S3 [file rsos160072supp3.tif]
