# Supplementary material for: New information on the braincase and inner ear of Euparkeria capensis Broom: implications for diapsid and archosaur evolution
Source: R Soc Open Sci. 2016 Jul 13;3(7):160072. doi: 10.1098/rsos.160072 (PMC4968458; doi:10.1098/rsos.160072)
Supplement: SUPPLEMENTARY FIGURES CAPTIONS [file rsos160072supp4.pdf]

SUPPLEMENTARY FIGURES CAPTIONS

Figure S1. Stereopairs of braincase of SAM-PK-7696 in (A) anterior, (B) posterior, (C) dorsal, (D) ventral, (E) right lateral, and (F) left lateral view.

Figure S2. Stereopairs of braincase of UMZC T.692 in (A) left lateral, (B) posterior, and (C) dorsal view, and separated right hand wall of braincase in (D) medial and (E) lateral view. Scale below A applies to A, B and C, and that below E applies to D and E.

Figure S3. Stereopairs of braincase of SAM-PK-5867 in (A) posterior, (B) posteroventral and (C) right lateral view.
